# Non-genetic risk and protective factors and biomarkers for neurological disorders: a meta-umbrella systematic review of umbrella reviews

**DOI:** 10.1186/s12916-020-01873-7

**Published:** 2021-01-13

**Authors:** Alexios-Fotios A. Mentis, Efthimios Dardiotis, Vasiliki Efthymiou, George P. Chrousos

**Affiliations:** 1 Public Health Laboratories, Hellenic Pasteur Institute, Athens, Greece; and, Department of Neurology, University Hospital of Larissa, University of Thessaly, Larissa, Greece; 2 Department of Neurology, University Hospital of Larissa, University of Thessaly, Larissa, Greece; 3 University Research Institute of Maternal and Child Health and Precision Medicine, National and Kapodistrian University of Athens, Athens, Greece; 4 University Research Institute of Maternal and Child Health and Precision Medicine, and UNESCO Chair on Adolescent Health Care, National and Kapodistrian University of Athens, Athens, Greece

**Keywords:** Brain diseases, Nervous system diseases, Meta-analysis, Systematic review, Umbrella review, Risk factors, Protective factors

## Abstract

**Background:**

The etiologies of chronic neurological diseases, which heavily contribute to global disease burden, remain far from elucidated. Despite available umbrella reviews on single contributing factors or diseases, no study has systematically captured non-purely genetic risk and/or protective factors for chronic neurological diseases.

**Methods:**

We performed a systematic analysis of umbrella reviews (*meta-umbrella*) published until September 20th, 2018, using broad search terms in MEDLINE, SCOPUS, Web of Science, Cochrane Database of Systematic Reviews, Cumulative Index to Nursing and Allied Health Literature, ProQuest Dissertations & Theses, JBI Database of Systematic Reviews and Implementation Reports, DARE, and PROSPERO. The PRISMA guidelines were followed for this study. Reference lists of the identified umbrella reviews were also screened, and the methodological details were assessed using the AMSTAR tool. For each non-purely genetic factor association, random effects summary effect size, 95% confidence and prediction intervals, and significance and heterogeneity levels facilitated the assessment of the credibility of the epidemiological evidence identified.

**Results:**

We identified 2797 potentially relevant reviews, and 14 umbrella reviews (203 unique meta-analyses) were eligible. The median number of primary studies per meta-analysis was 7 (interquartile range (IQR) 7) and that of participants was 8873 (IQR 36,394). The search yielded 115 distinctly named non-genetic risk and protective factors with a significant association, with various strengths of evidence. Mediterranean diet was associated with lower risk of dementia, Alzheimer disease (AD), cognitive impairment, stroke, and neurodegenerative diseases in general. In Parkinson disease (PD) and AD/dementia, coffee consumption, and physical activity were protective factors. Low serum uric acid levels were associated with increased risk of PD. Smoking was associated with elevated risk of multiple sclerosis and dementia but lower risk of PD, while hypertension was associated with lower risk of PD but higher risk of dementia. Chronic occupational exposure to lead was associated with higher risk of amyotrophic lateral sclerosis. Late-life depression was associated with higher risk of AD and any form of dementia.

**Conclusions:**

We identified several non-genetic risk and protective factors for various neurological diseases relevant to preventive clinical neurology, health policy, and lifestyle counseling. Our findings could offer new perspectives in secondary research (*meta-research*).

## Background

Chronic non-communicable neurological diseases, such as Alzheimer disease (AD) and movement disorders, and neuro-inflammatory diseases [e.g., multiple sclerosis (MS)], among others, represent the leading and second-leading causes of disability and mortality worldwide, respectively [1, 2]. Nowadays, because of improvements in quality of life, population growth, and longevity, a higher proportion of people are reaching ages harboring the highest prevalence of neurological diseases [3]. Furthermore, despite the high contribution of these nosological entities to the Global Burden of Disease, there has been only partial elucidation of their etiologies (for a discussion, which extends this study’ aims on the potential *communicable* etiology of such non-communicable diseases, see [4]). This important gap lies in contrast to other diseases, such as cancer and cardiovascular disorders, where research efforts have been far more prolific. Most published findings suggest an interplay of genetic predisposition risk and protective factors for neurological disorders [5–7] (a term used herein interchangeably with the term neurological disease and neurological condition [for a discussion on their differences, see [8])], while, in parallel, major health and public policy reports provide annual updates assessing how much major risk factors contribute to the chronic burden of neurological diseases and have addressed urgent calls for action on such disorders, including mitigation of risky lifestyle factors [9–11].

The contribution of several non-modifiable genetic factors to neurological disorders has been examined to some extent. Thus, studies on single-nucleotide polymorphisms (SNPs) and genome (GWAS) and transcriptome-wide association studies have revealed numerous possibly related SNPs and mechanistic clues [12–14]. Field synopses, as well as meta-analyses of GWAS, have also been reported (e.g., PDGene, AlzGene, and AlsGene) [15–23]. Nonetheless, as shown by updated study designs (*phenome/ exposome/ environment-wide association studies*), relevant statistical tools (e.g., *mediation* and *multivariable Mendelian randomization*), and epidemiological approaches (e.g., *triangulation* approaches [24]), this interplay has become even more complex because of the many confounders [25–32]. For instance, aging appears to be a principal risk factor for neurodegenerative diseases. However, the aging process encompasses the (patho) physiological unfolding of life, as well as the contribution of genetic and lifestyle determinants [33].

The contribution of environmental factors to neurological disorders is in many cases modifiable (further discussed in [34]). These factors contribute significantly to chronic non-communicable disease progression; notably, around 25% of global deaths may be due to threatening changes in our environment [35]. In a similar way, around 60% of cardiovascular mortality, a principal contributor to total mortality, can be attributed to eight major preventable risk factors [36]. Thus, obtaining solid evidence on these modifiable factors is crucial for evidence-based clinical neurological counseling, health promotion strategies, and patient risk awareness, addressed either at high-risk individuals or at the population at large [37]. Interestingly, recent attempts using health insurance datasets have been made to co-examine the contributions of genetic and non-genetic (also described as environmental) factors on the same individual’s clinical phenotype [38].

Umbrella reviews, the number of which has been blossoming since the first endorsement of this review type by Cochrane in 2009, are structured through the systematic retrieval, collection, and assessment of information and tested for consistency of evidence of previously published systematic reviews and meta-analyses [37, 39], as initially discussed in [40]. The end result is to collate compelling evidence into a single, informative review offering a broad view of a certain field to the medical community, aiming to cover knowledge gaps [41]. In particular, an umbrella review facilitates the comparison between different meta-analyses by repeating the analyses of the latter in a so-called *uniform approach for all factors*, considering the expected variability in their quality, focus-of-interest, and degree of evidence reliability [37, 42]. The methodology of meta-analyses appears to have increased statistical power, and umbrella reviews are frequently employed to help synthesize the available literature to guide both clinical care and public health policies. Collectively, umbrella reviews lie at the top of the hierarchy in the evaluation of evidence [2].

Several umbrella reviews have analyzed the risk and protective factors for a certain disease or condition, or the effects of some of these factors on multiple health outcomes, based on meta-analyses or Mendelian randomization studies [43], diagnostic criteria, and screening tools [44], diagnostic accuracy studies [45], therapeutic interventions [46], clinical efficacy of drugs [47], and/or interactions between genetic and environmental factors [48]. With regard to brain health, several umbrella reviews have analyzed meta-analyses and systematic reviews reporting an association between environmental factors and a single non-communicable neurological disorder (e.g., the risk factors for MS) [49–51], while others have studied the roles of a single factor (e.g., vitamin D levels) into multiple health outcomes, including neurological disorders [52].

Of note, it is increasingly recognized that the factors in question may exert distinct, even opposite effects in different neurological disorders, and that the evidence and/or the *credibility* of this epidemiological evidence (for a discussion on this term, see [53]) may be different across distinct neurological disorders [54]. Thus, there is an urgent need to identify, compare, and contrast—common (i.e., found in more than one disease under consideration) versus disease-specific, frequent versus rare, similar versus opposite—risk as well as protective factors of neurological disorders.

Such an *overarching* or encompassing study may be clinically important, as it will provide the opportunity to assess neurological disorders with shared versus specific risk and protective factors, which an umbrella review of a single risk factor is, by its design, not capable to address. Thus, performing a *systematic review of umbrella reviews*—an approach we wish to call *meta-umbrella*—may save an enormous amount of time compared to obtaining and reading the large number of individual umbrella reviews.

Hence, the aim of this *meta-umbrella* review was as follows: (a) to summarize and critically review, in a systematic manner, the available data and identify the gaps presented in previous umbrella reviews regarding risk and protective factors for the sum of chronic non-communicable neurological disorders analyzed, in order to offer an *overarching* field-wide overview; (b) to assess the *cream-of-the-cream* evidence and, more broadly, the levels of evidence spanning the last decades and to highlight factors that have displayed the most persuasive evidence of an association, from an evidence-based lens, while, in parallel to this, detecting points that the original studies might have missed, as well potential negative aspects of such studies; (c) to introduce an additional type of methodology and study design in the blossoming *meta-research* field, which, as a novel approach, could be applied to other disease categories (e.g., cardiovascular or neoplastic diseases) in the future; (d) to equip clinicians, preventive medicine specialists, and policymakers with solid evidence for performing their health care-related tasks, and for creating policy-formulating guidelines to address neurological disorders with shared risk and protective factors; (e) to provide a thorough discussion on the mechanisms underpinning the association of these risk and protective factors with neurological disorders, in order to address research gaps, at both translational and clinical levels, regarding how these factors interact with the pathogenesis of neurological diseases. Similarly to other evidence-based approaches for preventing certain neurological disorders [55], the ultimate goal of this study was to identify and address (e.g., through behavioral modifications) the risk and protective factors in question (e.g., obesity) early on, from midlife, and even early adulthood, but also from childhood and adolescence, in light of the Developmental Origins of Disease approach [56–58], in order to help reduce the incidence and, hence, the prevalence of neurological diseases (within the context of primary prevention).

## Methods

### Structure of Meta-umbrella review

We conducted a systematic review of umbrella reviews, which we call a *meta-umbrella review*, without any advance registration of the review’s goals or protocol in a relevant database. Our systematic search of the literature demonstrated that published umbrella reviews follow two approaches—a review of known risk factors for a single clinical outcome and a review of the relation between a single risk factor and multiple clinical outcomes (for example, [51] and [52]). With regard to evidence from observational associations between chronic non-communicable neurological disorders and known genetic risk and/or protective factors or a review of the relations between a single risk or protective factor with multiple neurological disorders, we retrieved data from published systematic (i.e., not-narrative) umbrella reviews of systematic reviews and meta-analyses. Such umbrella reviews (e.g., in [49]) were conducted using standardized methods (reviewed in [37]). Following guidelines for conducting umbrella reviews, we have critically assessed and comprehensively presented the quantitative data of the meta-analyses conducted in published umbrella reviews [37, 41, 59, 60], while the qualitative results of systematic reviews discussed in these umbrella reviews were not further considered. Herein, we applied a *pragmatic approach* similar to the one used in previous umbrella reviews, when the study design or/and sample sizes are missing in the meta-analysis, a condition otherwise essential for estimating excess of bias or conducting relevant subgroup analyses. Accordingly, we only considered the definitions of risk and protective factors used in the umbrella reviews and the information solely available therein (for further discussion of this concept, see [37]).

### Search strategy and eligibility criteria

Using a standardized search strategy (*generic* version is presented in Appendix 1), which was specified according to each database (data not shown), we systematically explored the following databases: MEDLINE, SCOPUS, Web of Science, Cochrane Database of Systematic Reviews, Cumulative Index to Nursing and Allied Health Literature, ProQuest Dissertations & Theses (in order to take into account gray literature), JBI Database of Systematic Reviews and Implementation Reports, DARE, and PROSPERO, registered up to September 20th, 2018, in order to identify umbrella reviews analyzing associations of non-purely genetic risk and protective factors with multiple chronic neurological disorders or umbrella reviews of single such factors with multiple clinical outcomes, in alignment with the WHO definition of neurological disorders [61] (Appendix 2).

We used broad search terms (umbrella review$ OR umbrella review$.ti,ab.) and other relevant keywords (stroke*, Alzheimer disease or dementia*, multiple sclerosis*, headache*, amyotrophic lateral sclerosis*, Parkinson disease, neurolog$) OR (multiple outcomes). Furthermore, harnessing a *snowball* procedure, citations and reference lists of the umbrella reviews were systematically screened, following the example of other studies [62].

**Fig. 1:**
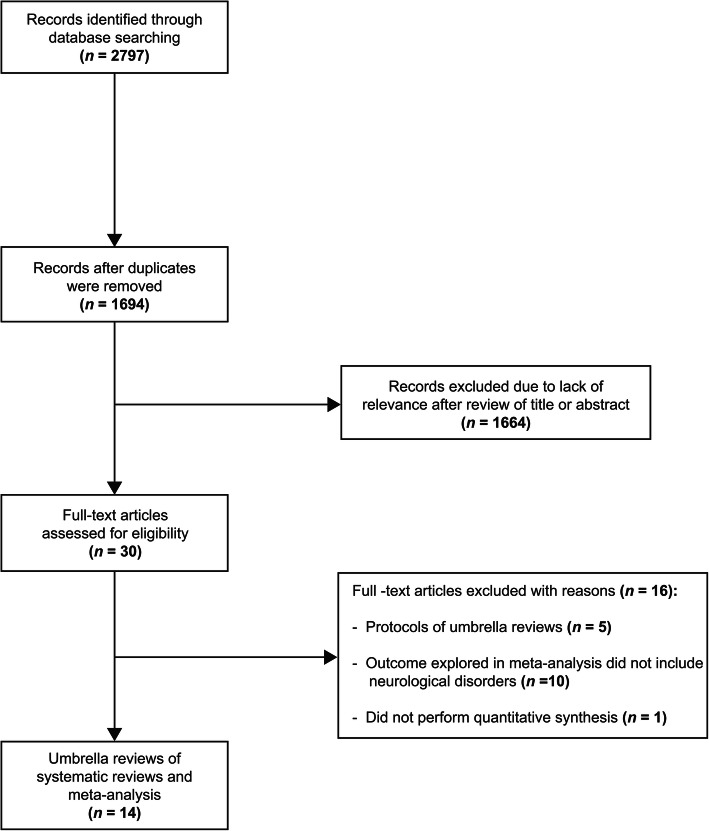
PRISMA flowchart

Based on predefined exclusion and inclusion criteria, four reviewers (AFAM, ED, VE, and GPC) independently conducted a three-step evaluation of the title, abstract, and full text of the papers (Fig. 1—*Preferred Reporting Items for Systematic Reviews and Meta-analyses* (PRISMA) flowchart of the current *meta-umbrella* systematic review), and any discrepancy between the four investigators was resolved by consensus. We only opted to retain umbrella reviews that investigated the association of environmental and non-purely genetic risk or protective factors with all types of chronic neurological disorders, or umbrella reviews of a single risk factor with multiple clinical outcomes. There was no selective inclusion of umbrella reviews reporting only on systematic reviews of observational (in general) or prospective studies (in particular), or those reporting on clinical (randomized) trials, or those of Mendelian randomization studies (with the exception of purely genetic factors). Restrictions regarding language were not applied in the search strings or in eligible study selection. Due to previous concerns that completely distinguishing genetic from environmental risk and protective factors could be deceptive, we followed these studies’ *pragmatic approach* to refer to *non-purely genetic factors* with regard to protective and risk *factors.* This approach is defined as one applying the definitions of the papers that are included in the review process, instead of creating new definitions [34].

Exclusion of studies took place if any among the following criteria was relevant: (a) referring to a protocol for umbrella reviews, and not an umbrella review study per se; (b) the examined factor(s) was (were) deemed as pure genetic factor(s) or genetic biomarker(s) (because these factors are non-modifiable, and because different analytical methods and criteria are required for umbrella reviews of genetic variables); (c) the factor(s) or neurological conditions referred to mostly neurosurgical and/or brain traumatic disorders (e.g., brain injury or neuro-oncological diseases), or neurological conditions with a subjective component (e.g., pain); (d) the studies consisted of umbrella reviews assessing clinical outcomes (e.g., decline, impairment, relapse or remission) of neurological disorders, the severity of their clinical presentation, and the effects of a treatment or an intervention for a neurological disease; and (e) umbrella reviews referring exclusively to studies in animals. Nonetheless, following the methodology of previous studies [63], we did not exclude umbrella reviews that evaluated cross-sectional studies, as the latter, although offering valuable associations, may not allow causality inference.

If multiple meta-analyses on the same research question were eligible for inclusion, then they were all presented with the indication of *overlapping*. Considering that the umbrella reviews’ structure follows that of standard systematic reviews, its quality and integrity were validated using PRISMA [64] (Additional file 1: PRISMA Checklist).

### Data extraction

Four investigators (AM, ED, VE, and GPC), in groups of two, performed the data extraction from the studies. The first author, journal, and the year of publication of each eligible article were documented. Additionally, we recorded the risk and protective factors, biomarkers, and the chronic, non-communicable neurological conditions analyzed, number of studies reviewed, study-specific risk estimates [i.e., odds ratio (OR), hazard ratio, risk ratio (RR), or other pertinent effect size], alongside their corresponding confidence interval (CI), and the number of participants in each study. We, also, reviewed whether the included studies performed any quality control over the reviews and meta-analyses used.

For data extracted from studies where one non-purely genetic risk or protective factor or biomarker was reviewed en face of multiple health outcomes, we extracted only data that were relevant to neurological diseases.

As no standard criteria exist to assess the quality of umbrella reviews, we used the current expert recommendations and the *A Measurement Tool to Assess Systematic Reviews* (AMSTAR) method to assess the quality of reviews [37, 65] (since no major differences have been identified compared to using AMSTAR-2, to our knowledge) [66–68]. Notwithstanding its limitations (including, among others, a heavy dependence on the so-called *reporting* quality and not the *methodological* one, as well as the lack of focus on the sample size and the design of single studies, as discussed in [69, 70]), AMSTAR is a tool that applies dichotomous scoring (i.e., 0 or 1) for 11 items (e.g., publication bias assessment) to evaluate systematic reviews, notably to assess: (a) the quality of their methodology, (b) if the search strategy is a thorough one; (c) how much prone to bias is every systematic review; and (d) how appropriate are the statistical tools applied for the meta-analysis [63]. The AMSTAR method was applied by completing a checklist with specific questions on these systematic reviews. If graded between 8 and 11, 4–7, or 0–3, then AMSTAR scores were deemed of high, medium, and low quality, respectively.

### Data analysis

We performed a descriptive analysis of umbrella reviews. We specifically reviewed and recorded the summary effect size and its 95% CI using the random effects model of the meta-analyses presented in these umbrella reviews. This model was opted instead of the fixed effects model because (a) the random effects model considers the potential heterogeneity of results between studies and (b) because the classes of evidence (as below) that were relevant to our selection criteria were based on values in random-effects model [71, 72]. We also recorded the 95% prediction interval for each estimate, a feature that helps determine the uncertainty with regard to an effect expected in a new study, in which the same association was assessed, by considering the between-study heterogeneity [73]. In addition, we recorded the *I*^2^ metric, used to analyze any inter-study heterogeneity [74]. The *I*^2^ ranges, which estimate the proportion of inter-study variance over the sum of the intra- and inter-study variances, were between 0 and 100% [74]. Values > 50% or > 75% represent large or very large heterogeneity, respectively (as in [49–51]). We also reported whether small-study effects were described, i.e., whether smaller studies exaggerated a reported effect as opposed to larger studies using Egger’s regression asymmetry test [75], when applicable. The underlying rationale is that both large heterogeneity and plausible reporting of excess significance bias reduce the accuracy of evidence of a contributing factor, regardless of the *p* value and effect size [34].

### Assessment of credibility of epidemiological evidence

Regarding the association between a risk or protective factor or biomarker and a neurological disorder, we recorded the conclusions of each umbrella review according to the sub-categories of analyses that were based on the meta-analyses reviewed. Then, we assessed the strength of the association between risk and protective factors and biomarkers, from the one side, and neurological disorders from the other.

In general, in our *meta-umbrella* approach, we followed a *pragmatic approach*, and we present each classification of credibility for evidence that was applied by separate studies in Table 4.

When a pragmatic approach was not possible (mostly due to lack of available relevant data), we assessed the strength using the criteria for the assessment of the credibility of epidemiological evidence based on previous umbrella reviews [49–51, 76–82]. In doing so, we applied the above studies’ criteria and definitions on the following classes of evidence, which appear to consider both *p* value and the prediction intervals. This was performed in alignment with recent expert recommendations that these criteria contribute to classify the accuracy level of evidence in a standardized and objective manner in umbrella reviews [54], and, as a corollary, in the systematic reviews of umbrella reviews (i.e., meta-umbrella). Therefore, based on the previous expert recommendations and already conducted relevant umbrella review studies, we categorized the observed associations into classes of evidence (i.e., class I–IV) based on whether each association was convincing, by using a combination of the following criteria (which take into account both the *p* value and the magnitude of the association): (a) levels of significance of the random effects model (*P* ≤ 0.05, *P* ≤ 0.001, and *P* < 10^− 6^); (b) level of significance of the largest component study (*P* < 0.05); (c) inclusion of > 1000 participants (or number of participants greater than 20,000 with regard to continuous outcomes); (d) absence of considerable heterogeneity (*I*^2^ < 50%); (e) lack of evidence of either small-study effect (*P* > 0.10) or excess significance (*P* > 0.10); and (f) 95% prediction interval excluded the null value, as reported in previous studies [49–51, 76–82]. Of note, the variables applied in the above criteria are continuous, whereas the chosen cut-off points are arbitrarily selected [37].

In this context, the following categories of evidence were applied: (a) class I (*convincing*): statistical significance based on the random effects model with *P* < 10^− 6^, > 1000 cases or deaths (or number of participants greater than 20,000 with regard to continuous outcomes), the largest component study reporting statistically significant effect (*P* < 0.05), 95% prediction interval excluding the null value, without large inter-study heterogeneity (*I*^2^ < 50%), no evidence of excess of significance (*P* > 0.10), and absence of evidence of small-study effect (*P* > 0.10); (b) class II (*highly suggestive*): statistical significance with *P* < 10^− 6^, > 1000 cases or deaths (or number of participants greater than 20,000 with regard to continuous outcomes), and the largest component of the study reported statistically significant effect (*P* < 0.05); (c) class III (*suggestive*): statistical significance with *P* < 10^− 3^, > 1000 cases or deaths (or number of participants greater than 20,000 with regard to continuous outcomes); (d) class IV (*weak*): the remaining statistically significant associations with *P* < 0.05; and whereas (e) *non-significant*: associations with *P* ≥ 0.05 (reviewed also in [60]). Finally, the statistical analyses were retrieved from the umbrella reviews (when available).

### Data availability statement

Data sharing is not pertinent or applicable to this study given that no production or analysis of data sets took place during this study.

## Results

### Search results

The electronic search of the relevant databases yielded 2797 potentially relevant reviews; of these, 14 umbrella reviews fulfilled the eligibility criteria and were included in the study (Fig. 1) [49–52, 73, 76–79, 81–85]. No additional studies were located using the *snowball* procedure.

Regarding the number of studies addressing one disease as an outcome or multiple disease processes, the following distinction was observed: four were identified as studies addressing multiple risk factors for a single neurological condition [49–51, 78], whereas 10 were identified as addressing one risk factor for multiple health outcomes, including neurological conditions [52, 73, 76, 77, 79, 81–85]. Also, in the single umbrella review comparing meta-analyses of observational studies with randomized controlled trials, there was no discordant direction of results with regard to neurological disorders [52] (data not shown in a diagram).

The 14 umbrella reviews corresponded to 203 unique meta-analyses of factors with marked association with 12 neurological conditions [AD, Parkinson disease (PD), dementia, vascular dementia, cognitive impairment/disorders, MS, amyotrophic lateral sclerosis (ALS), neuromyelitis optica, glioma, neurodegenerative diseases, diabetic peripheral neuropathy, and stroke].

The minimum and maximum number of studies included in the systematic reviews and meta-analyses of any of the risk or protective factors or biomarkers was 1 and 67, respectively. In Additional file 2, Table S1 describes the factors, neurological conditions, and overall results of the selected studies, including the detailed data extraction. The median number of primary studies per meta-analysis was 7 [interquartile range (IQR) 7], and the median number of participants was 8873 (IQR 36,394). The 203 meta-analyses investigated multiple non-purely genetic factors, such as diet, drugs, medical history, comorbid disease, psychological/behavioral traits, and habits and exposure to toxic environments. In general, when multiple umbrella reviews reported the same meta-analysis, all these overlapping meta-analyses were presented. Overall, we summarized 115 distinctly named factors associated with these neurological conditions.

In Additional file 2, Table S2 shows the methodological quality of the selected umbrella reviews, as assessed using the AMSTAR criteria [65]. The total AMSTAR score of the reviews ranged from 7 to 9 points, while the mean score was 8.0 points, and the standard deviation was 0.39. Questions most frequently satisfied were questions 2–4 (related to duplicate study selection and data extraction, search comprehensiveness, and inclusion criteria). Other aspects of the AMSTAR score commonly satisfied by the reviews included questions 6–9 (related to the characteristics and scientific quality of the included studies, along with appropriateness of the methods used to combine the studies). Questions 1, 11 (related to a priori study design and conflict of interest), and 5 (pertaining to reporting and provision of included and excluded list of studies) were the least frequently satisfied.

### Commonly observed protective and risk factors of neurological conditions

After comparing all data, Table 1 summarizes the protective factors for neurological conditions. Mediterranean diet was a common protective factor for dementia, AD, cognitive impairment, mild cognitive impairment incidence, neurodegenerative diseases, and stroke. Bone mineral density in lumbar spine, femoral neck, hip, and serum vitamin B_12_ were associated with a reduced risk of developing MS and PD. Vitamin E and non-aspirin nonsteroidal anti-inflammatory drugs (NSAIDs) were protective factors for PD and AD. Serum vitamin D levels were associated with a lower risk of MS, AD, PD, cognition, and stroke. Physical activity and alcohol intake were associated with a reduced risk of developing AD, PD, vascular dementia, and all types of dementia (Table 1). High caffeine and coffee consumption were associated with a lower risk of PD, AD, and cognitive impairment/disorders, or PD, AD, stroke, glioma, and cognitive decline, respectively. Statins were found to be protective against PD, AD, and dementia. Furthermore, antihypertensive drugs exerted a protective effect against vascular dementia and all types of dementia.

**Table 1:** Protective factors that are common in non-communicable neurological disorders (i.e., found in more than one disease under consideration)

Factor	Effect size metric	Effect size (95% CI)	Neurological condition (systematic review/meta-analysis)	Level of evidence	Umbrella review study
Mediterranean diet	RR	0.83 (0.75–0.93)	Mild cognitive impairment incidence (Wu, 2017) ^@^	IV	Galbete, 2018 [84]; Dinu, 2018^#^ [81]
	RR	0.60 (0.48–0.77)	Alzheimer disease (Wu, 2017) ^@^	IV	Galbete, 2018 [84]; Dinu, 2018^#^ [81]
	RR	0.69 (0.57–0.84)	Mild cognitive impairment incidence/Dementia (Cao, 2016) ^@^	IV	Galbete, 2018 [84]
	HR	0.73 (0.56–0.96)	Mild cognitive impairment incidence (Singh, 2014) ^@^	IV	Galbete, 2018 [84]
	RR	0.64 (0.46–0.89)	Alzheimer disease incidence (Singh, 2014) ^@^	IV	Galbete, 2018 [84]
	RR	0.84 (0.74–0.95)	Stroke (Psaltopoulou, 2013; Cohort studies) ^@^	IV	Galbete, 2018 [84] Dinu, 2018^#^ [81]
	RR	0.20 (0.10–0.41)	Stroke (Psaltopoulou, 2013; Case-control studies) ^@^	Weak	Dinu, 2018 [81]
	RR	0.83 (0.66–1.06)	Stroke (Psaltopoulou, 2013; Cross-sectional studies) ^@^	No evidence	Dinu, 2018 [81]
	RR	0.76 (0.60–0.96)	Stroke (Grosso, 2014; Cohort studies) ^@^	Weak	Dinu, 2018 [81]
	RR	0.87 (0.81–0.94)	Neurodegenerative diseases (Sofi, 2014) ^@^	Convincing	Dinu, 2018 [81]
	RR	0.79 (0.70–0.90)	Neurodegenerative diseases (Wu, 2017) ^@^	Highly suggestive	Dinu, 2018 [81]
	RR	0.72 (0.58–0.88)	Cognitive impairment (Psaltopoulou, 2013; Cohort studies) ^@^	Suggestive	Dinu, 2018 [81]
	HR	0.67 (0.55–0.81)	Cognitive impairment (Singh, 2014; High-vs.-low MeDi score) ^@^	Suggestive	Dinu, 2018 [81]
	HR	0.92 (0.88–0.97)	Cognitive impairment (Singh, 2014; 1-point increase in MeDi score) ^@^	Suggestive	Dinu, 2018 [81]
	RR	0.31 (0.16–0.59)	Cognitive impairment (Psaltopoulou, 2014; Case-control) ^@^	Weak	Dinu, 2018 [81]
	RR	0.83 (0.75–0.93)	Cognitive impairment (Wu, 2017) ^@^	Highly suggestive	Dinu, 2018 [81]
	RR	0.69 (0.57–0.84)	Dementia	Convincing	Dinu, 2018 [81]
	RR	0.64 (0.47–0.86)	Stroke (Grosso, 2014; Randomized trials) ^@^	Weak	Dinu, 2018 [81]
Caffeine	RR	0.67 (0.57–0.80)	Parkinson disease	Probable	Grosso, 2017 [76]
	N/SP	0.78 (0.50–1.22)	Alzheimer disease	Limited	Grosso, 2017 [76]
	N/SP	0.79 (0.61–1.04)	Cognitive impairment	Limited	Grosso, 2017 [76]
	N/SP	0.82 (0.67–1.01)	Cognitive disorders	Possible	Grosso, 2017 [76]
Coffee consumption	RR	0.97 (0.85–1.11)	Cognitive decline	6^*^	Poole, 2017 [79]
	RR	0.96 (0.83–1.11)	Stroke	8^*^	Poole, 2017 [79]
	RR	0.98 (0.79–1.23)	Glioma	5^*^	Poole, 2017 [79]
	RR	0.64 (0.53–0.76)	Parkinson disease (Qi, 2014) ^@^	5^*^	Poole, 2017 [79]
	RR	0.64 (0.53–0.77)	Parkinson disease (Noyce, 2012) ^@^	7^*^	Poole, 2017 [79]
	RR	0.73 (0.54–0.99)	Alzheimer disease (Barranco Quintana, 2017) ^@^	3^*^	Poole, 2017 [79]
	RR	0.70 (0.56–0.88)	Parkinson disease (Hernan, 2002) ^@^	Probable	Grosso, 2017 [76]
	RR	0.73 (0.55–0.97)	Alzheimer disease (Liu, 2016) ^@^	Possible	Grosso, 2017 [76]
	RR	0.67 (0.58–0.76)	Parkinson disease (Noyce, 2012)^@^	III	Bellou, 2016 [50]
Alcohol intake	RR	0.74 (0.61–0.91)	Dementia	Weak	Bellou, 2017 [49]
	RR	0.75 (0.57–0.98)	Vascular dementia	Weak	Bellou, 2017 [49]
	RR	0.72 (0.61–0.86)	Alzheimer disease	Weak	Bellou, 2017 [49]
	RR	0.75 (0.66–0.85)	Parkinson disease	III	Bellou, 2016 [50]
Physical activity	RR	0.76 (0.66–0.86)	Dementia	Suggestive	Bellou, 2017 [49]
	RR	0.62 (0.42–0.92)	Vascular dementia	Weak	Bellou, 2017 [49]
	HR	0.66 (0.57–0.78)	Parkinson disease	I	Bellou, 2016 [50]
	HR	0.62 (0.52–0.72)	Alzheimer disease	Highly suggestive	Bellou, 2017 [49]
Bone mineral density in femoral neck	OR	0.36 (0.21–0.61)	Multiple sclerosis	Weak	Belbasis, 2015 [18, 51]
	OR	0.25 (0.09–0.66)	Parkinson disease	IV	Bellou, 2016 [50]
Bone mineral density in hip	OR	0.33 (0.18–0.60)	Multiple sclerosis	Weak	Belbasis, 2015 [18, 51]
	OR	0.55 (0.38–0.80)	Parkinson disease	IV	Bellou, 2016 [50]
Bone mineral density in lumbar spine	OR	0.34 (0.24–0.50)	Multiple sclerosis	Weak	Belbasis, 2015 [18, 51]
	OR	0.29 (0.16–0.54)	Parkinson disease	IV	Bellou, 2016 [50]
Serum vitamin D	SMD to RR	0.08 (0.01–0.63)	Alzheimer disease	No conclusion	Theodoratou, 2014 [52]
	OR	0.42 (0.34–0.53)	Cognition	Suggestive	Theodoratou, 2014 [52]
	RR	0.61 (0.50–0.75)	Stroke	Suggestive	Theodoratou, 2014 [52]
	HR	0.66 (0.55–0.80)	Ischemic stroke	Suggestive	Theodoratou, 2014 [52]
	OR	0.52 (0.44–0.61)	Ischemic stroke	Suggestive	Theodoratou, 2014 [52]
	OR	0.16 (0.05–0.50)	Parkinson disease	IV	Bellou, 2016 [50]
	OR	0.44 (0.24–0.70)	Multiple sclerosis	Weak	Belbasis, 2015 [18, 51]
Serum vitamin B_12_	OR	0.64 (0.44–0.93)	Multiple sclerosis	Suggestive	Belbasis, 2015 [18, 51]
	OR	0.50 (0.40–0.63)	Parkinson disease	IV	Bellou, 2016 [50]
Vitamin E dietary intake	RR	0.80 (0.67–0.95)	Alzheimer disease	Weak	Bellou, 2017 [49]
	OR	0.81 (0.67–0.98)	Parkinson disease	IV	Bellou, 2016 [50]
Statins	RR	0.83 (0.76–0.91)	Dementia	Suggestive	Bellou, 2017 [49]
	RR	0.72 (0.59–0.89)	Alzheimer disease	Weak	Bellou, 2017 [49]
	RR	0.77 (0.64–0.92)	Parkinson disease	IV	Bellou, 2016 [50]
Antihypertensive drugs	HR	0.84 (0.75–0.94)	Dementia	Weak	Bellou, 2017 [49]
	RR	0.64 (0.42–0.98)	Vascular dementia	Weak	Bellou, 2017 [49]
Non-aspirin NSAIDS	RR	0.85 (0.77–0.94)	Parkinson disease	IV	Bellou, 2016 [50]
	RR	0.65 (0.49–0.86)	Alzheimer disease	Weak	Bellou, 2017 [49]

*Abbreviations*: *N/A* not available, *OR* odds ratio, *RR* relative risk, *HR* hazard ratio, *SMD* standardized mean difference, *N/SP* not specified (either OR or RR), *95% CI* 95% confidence interval

^*^According to AMSTAR classification. ^#^ Overlapping studies. ^@^These refer to metrics (e.g., RRs, HRs) of the original systematic reviews included in the umbrella reviews. The full citations of these original systematic reviews/meta-analyses are included in the corresponding umbrella reviews

Note: In this table, only statistically significant risk factors that appeared in more than two studies are included

Table 2 presents the common risk factors of neurological disorders, after comparing all data. Based on class III/IV evidence, exposure to farming, pesticides, and head injuries were risk factors for ALS and PD, while mild traumatic brain injury and high midlife body mass index (BMI) were risk factors for AD and dementia (Table 2). Exposure to low-frequency electromagnetic fields was a risk factor for ALS and AD. Additionally, exposure to organic solvents (class IV evidence) was a risk factor for PD and MS. Type 2 diabetes mellitus, depression at any age/stage, late-life depression, and low education were risk factors for AD, vascular dementia, and all types of dementia.

**Table 2:** Risk factors that are common in non-communicable neurological disorders (i.e., found in more than one disease under consideration)

Factor	Metric	Effect size (95% CI)	Neurological condition	Level of evidence	Study
Farming	OR	1.42 (1.17–1.73)	Amyotrophic lateral sclerosis	Suggestive	Belbasis, 2016 [78]
	OR	1.30 (1.16–1.46)	Parkinson disease	III	Bellou, 2016 [50]
Pesticides	OR	1.62 (1.40–1.88)	Parkinson disease	III	Bellou, 2016 [50]
	OR	1.44 (1.22–1.70)	Amyotrophic lateral sclerosis	Weak	Belbasis, 2016 [78]
Type 2 diabetes mellitus	RR	1.54 (1.39–1.72)	Alzheimer disease	Convincing	Bellou, 2017 [49]
	RR	1.60 (1.43–1.79)	Dementia	Highly suggestive	Bellou, 2017 [49]
	RR	2.28 (1.94–2.66)	Vascular dementia	Convincing	Bellou, 2017 [49]
Low-frequency electromagnetic fields	OR	1.29 (1.03–1.62)	Amyotrophic lateral sclerosis	Weak	Belbasis, 2016 [78]
	RR	1.74 (1.37–2.21)	Alzheimer disease	Suggestive	Bellou, 2017 [49]
Organic solvents	OR	1.54 (1.03–2.29)	Multiple sclerosis	Weak	Belbasis, 2016 [78]
	OR	1.22 (1.01–1.47)	Parkinson disease	IV	Bellou, 2016 [50]
Midlife BMI	RR	1.81 (1.22–2.69)	Alzheimer disease	Weak	Bellou, 2016 [50]
	RR	1.91 (1.40–2.62)	Dementia	Suggestive	Bellou, 2017 [49]
Head injury	OR	1.65 (1.09–2.51)	Amyotrophic lateral sclerosis	Suggestive	Belbasis, 2016 [78]
	OR	1.55 (1.33–1.81)	Parkinson disease	II	Bellou, 2016 [50]
Mild traumatic brain injury	OR	1.35 (1.01–1.78)	Dementia	Weak	Bellou, 2017 [49]
	OR	1.40 (1.03–1.90)	Alzheimer disease	Weak	Bellou, 2017 [49]
Depression at any age/stage	RR	1.99 (1.84–2.16)	Dementia	Convincing	Bellou, 2017 [49]
	RR	2.92 (1.87–4.56)	Vascular dementia	Weak	Bellou, 2017 [49]
	RR	1.77 (1.48–2.13)	Alzheimer disease	Highly suggestive	Bellou, 2017 [49]
Late-life depression	RR	1.85 (1.67–2.05)	Dementia	Convincing	Bellou, 2017 [49]
	OR	2.52 (1.77–3.59)	Vascular dementia	Weak	Bellou, 2017 [49]
	RR	1.65 (1.42–1.92)	Alzheimer disease	Convincing	Bellou, 2017 [49]
Low level of Education	RR	1.88 (1.51–2.33)	Dementia	Suggestive	Bellou, 2017 [49]
	RR	2.75 (2.19–3.45)	Vascular dementia	Weak	Bellou, 2017 [49]
	RR	1.82 (1.36–2.43)	Alzheimer disease	Suggestive	Bellou, 2017 [49]

*Abbreviations*: *BMI* body mass index, *OR* odds ratio, *RR* relative risk, *95% CI* 95% confidence interval

Note: In this table, only statistically significant risk factors that appeared in more than two studies were included

Three factors—tobacco smoking, hypertension, and serum uric acid—exerted a mixed (protective and risk) effect on neurological disorders (Table 3). On the one hand, tobacco smoking contributed to the development of MS and vascular/all types of dementia, while hypertension contributed to developing vascular dementia only. On the other hand, both tobacco smoking and hypertension were associated with a reduced risk of developing PD, according to class II and IV evidence, respectively. Individuals with high serum uric acid exhibited lower risk of developing PD, AD, ALS, MS, neuromyelitis optica, and dementia, but they had a higher risk of developing diabetic peripheral neuropathy and stroke mortality.

**Table 3:** Variables that are both risk and protective factors for non-communicable neurological disorders

Factor	Effect size metric	Effect size (95% CI)	Neurological condition	Level of evidence	Study
Smoking	RR	1.26 (1.05–1.50)	Vascular dementia	Weak	Bellou, 2017 [49]
	OR	1.52 (1.39–1.66)	Multiple sclerosis	Convincing	Belbasis, 2015 [18, 51]
	RR	0.64 (0.60–0.69)	Parkinson disease	II	Bellou, 2016 [50]
	RR	1.13 (1.05–1.22)	Dementia	Weak	Bellou, 2017 [49]
Hypertension	HR	1.59 (1.20–2.11)	Vascular dementia	Weak	Bellou, 2017 [49]
	RR	0.75 (0.61–0.90)	Parkinson disease	IV	Bellou, 2016 [50]
Serum uric acid	SMD to RR	0.58 (0.41–0.83)	Dementia	IV	Li, 2017 [86]
	SMD to RR	0.49 (0.27–0.87)	Multiple sclerosis	IV	Li, 2017 [86]
	RR	2.83 (2.13–3.76)	Diabetic peripheral neuropathy	IV	Li, 2017 [86]
	aRR	1.32 (1.23–1.41)	Stroke mortality	I	Li, 2017 [86]
	MD to OR	0.29 (0.11–0.76)	Alzheimer disease	IV	Li, 2017 [86]
	RR	0.65 (0.43–0.97)	Parkinson disease incidence	IV	Li, 2017 [86]
	OR	0.28 (0.14–0.57)	Multiple sclerosis	Weak	Belbasis, 2015 [18, 51]
	SMD to RR	0.22 (0.10–0.45)	Neuromyelitis optica	IV	Li, 2017 [86]
	Hedge’s to RR	0.21 (0.14–0.32)	Amyotrophic lateral sclerosis	IV	Li, 2017 [86]
	OR	0.39 (0.27–0.57)	Parkinson disease	II	Bellou, 2016 [50]

*Abbreviations*: *N/A* not available, *OR* odds ratio, *RR* relative risk, *HR* hazard ratio, *SMD* standardized mean difference, *aRR* adjusted relative risk, *MD* mean difference, *95% CI* 95% confidence interval

### Specific risk and protective factors of neurological conditions

In Additional file 2, Table S1 presents the specific risk and protective factors of neurological conditions, summarizing the findings based on class I–IV evidence. High β-carotene and n-3 fatty acid intake was significantly associated with a lower ALS risk. In contrast, exposure to lead and other heavy metals was significantly linked to a higher risk of developing ALS. A high level of exposure to welding, alpha-synuclein in cerebrospinal fluid (CSF), nigral volume, serum urate, retinal nerve fiber layer thickness, ibuprofen use, and calcium channel blockers, was associated with a lower risk of developing PD than did rural living, dairy product intake, constipation, head injury, hydrocarbon exposure, well water drinking, energy intake, carbohydrate intake, beta-blockers, and having anxiety or depression, all of which were associated with a higher risk of developing the disease (Additional file 2: Table S1).

Being overweight in late-life (based on BMI, assessed in a binary manner as obese vs. having normal weight) was significantly associated with a lower risk of developing dementia. Contrarily, a higher frequency of social contacts, loneliness, social participation, tooth loss, rheumatoid arthritis, benzodiazepine use, and atrial fibrillation were significantly associated with a higher risk of dementia. Diphtheria and tetanus vaccination, as well as a higher anti-Epstein-Barr virus (anti-EBV) IgG seronegativity, were significantly associated with a lower risk of developing MS. Conversely, anti-Epstein Barr nuclear antigen (anti-EBNA) IgG seropositivity, infectious mononucleosis, appendectomy at an age ≤ 20 years, EBV DNA in serum and mononuclear cells, tonsillectomy at an age ≤ 20 years, traumatic injury, anti-viral capsid antigen IgG seropositivity, chronic cerebrospinal venous insufficiency, serum homocysteine, and Chlamydia pneumoniae (DNA in CSF, intrathecal production of IgG) were significantly associated with a higher risk of developing MS.

Vitamin C, aspirin, NSAIDs, fish intake, agreeableness, conscientiousness, openness, and cancer were significantly associated with a lower risk of AD. Conversely, Chlamydia pneumonia infection, spirochetal infection, *Herpesviridae* infection, aluminum exposure, stroke, and neuroticism were significantly associated with a higher risk of developing AD (Additional file 2: Table S1).

## Discussion

Increasing accretion of data has led to recent calls for comprehensive, field-wise analyses of risk and protective factors for many human disorders [87]. Our study constitutes a *meta-umbrella* systematic review of non-genetic risk and protective factors linked to chronic neurological disorders published in earlier umbrella reviews and corresponding systematic reviews and meta-analyses. Accordingly, our study provides an encompassing and, in parallel, systematic (*overarching*) perspective on risk and protective factors and biomarkers, albeit not with quantitative approaches. In contrast, field-wide meta-analyses using quantitative approaches have assessed the *entire field of putative risk and protective factors* but for a specific disease, not an entire spectrum of diseases or body organ system (in this case, the central nervous system) [87].

Notably, following previous characterizations of umbrella reviews as *next-generation systematic reviews* [41], our approach can be conceived as a *third-generation systematic review*. It is an approach that aims to offer a new perspective of secondary research (*meta-research*), a field hallmarked by the need to provide the most integrated evidence possible, and in which several novel study designs have appeared during the last years, e.g., *series of systematic reviews and meta-analyses* in a single publication, where the *analytical unit* is the umbrella review study design [88]. Likewise, other attempts refer to *field-wide meta-analyses*, in which a meta-analysis of observational studies is conducted on the sum of risk factors under consideration [87], or to the synthesis of systematic reviews (e.g., in Neurology [89]), *systematic reviews of systematic reviews* [90], *overviews of systematic reviews* [91], *meta-reviews* [92], *systematic meta-reviews*, *comprehensive reviews* [93], *research-on-research* [94], and *meta-meta-analyses* [95, 96]; these are all terms and study designs that future meta-umbrella reviews should include in their search strategy. Therefore, our *meta-umbrella* review could represent another study design added to the armamentarium of *meta-research* [97].

Although our primary aim was to study the largest possible number of neurological conditions (which we expected to have been analyzed in umbrella reviews), we discovered that the umbrella reviews had studied only 12 neurological conditions. For instance, we could not find umbrella reviews on risk or protective factors for some common or major chronic neurological disorders such as migraine, headache, brain cancer, or epilepsy. Therefore, future umbrella reviews should be considered regarding the non-purely genetic risk factors of these conditions. Interestingly, almost all studies had focused on neurological disorders with high prevalence and in resource-rich countries, which could be indicative of the disproportionally lower number of publications regarding *meta-research* for global health neurology, namely neurological diseases of resource-poor countries (e.g., meningitis, neurocysticercosis), as well as for rare/orphan neurological disorders.

### Principal findings

We studied 115 distinctly named risk/protective factors with a marked association with chronic non-communicable neurological disorders, including biomarkers, habits, dietary factors, medical history or/and comorbid diseases, drugs, and exposure to toxic environmental agents. Fourteen factors exhibited a decreased risk for an extensive number of non-communicable neurological disorders, with these factors ranging in strength from class I to class IV. Below, we provide our insight into some of these findings.

Notably, the following associations appeared with class I evidence: (a) in neurodegenerative diseases, dementia, and AD, Mediterranean diet was a protective factor; (b) in MS, smoking, anti-EBNA IgG seropositivity, as well as infectious mononucleosis were risk factors; (c) in ALS, lead was a risk factor; (d) in PD, physical activity was protective, while constipation was a risk factor (although serious concerns were previously raised [50]); (e) in AD, late-life depression and type 2 diabetes mellitus were risk factors; (f) in dementia, depression at any age, life depression, frequency of social contacts, and benzodiazepine use were risk factors; (g) in vascular dementia, type 2 diabetes mellitus was a risk factor; (h) in stroke mortality, high uric acid levels were a risk factor.

While several risk and protective factors had class III and IV evidence of being significant in the occurrence of these neurological conditions, three of them, namely tobacco smoking, hypertension, and serum uric acid, exerted a mixed risk and beneficial effect. Based on the *I*^2^ metric, heterogeneity was present in published reports, and few studies were consistent with non-heterogeneous evidence when data had a prediction interval excluding the null.

With regard to dietary factors, we found substantial evidence highlighting the potential role of Mediterranean diet in lowering the risk of dementia, AD, cognitive impairment, neurodegenerative diseases, and stroke. Until now, several meta-analyses have reported quite solid evidence of the beneficial effect of Mediterranean diet in AD and other dementias, i.e., major categories of neurodegenerative disorders (for an example, see umbrella reviews [81] and [98]). However, discrepancies have been reported regarding the cardiovascular benefits of Mediterranean diet across socioeconomic groups [99]. Because of different reporting methods across studies in the field, development of standardized tools is imperative for the assessment of the effectiveness of Mediterranean diet in preventing cognitive impairment and neurodegenerative diseases. In a similar context, a recent study, consisting of a series of meta-analyses and including more than 130 million person-years of data from more than 240 original studies, presented quite solid evidence of low glycemic index food intake in stroke reduction [88].

In parallel, negative associations between coffee consumption and PD and AD [100] have been reported, with these findings being consistent across study designs and geographical settings. The biological mechanism(s) underlying this protective effect remain(s) unclear. For example, regular coffee intake enhances insulin sensitivity and, hence, reduces the risk of diabetes mellitus type 2, which itself is a strong risk factor for cognitive decline [101]. Also, recent meta-analyses, having considered the plausible roles of numerous modifiers, suggest that a 3.5-cup/day coffee intake is inversely associated with all-cause mortality, an association that has remained undiluted even after adjusting for major modifiers, such as aging, smoking, and alcohol consumption [102].

This systematic review of umbrella reviews revealed counterintuitively a significant association of low serum uric acid levels with a decreased risk of several neurological diseases (i.e., AD, PD, dementia, MS, neuromyelitis optica, and ALS), while diabetic peripheral neuropathy and stroke mortality were associated with an increased risk. Our credibility assessment revealed that, with the exception of PD (with class II evidence) and stroke mortality (with class I evidence), these significant associations were within class IV evidence [82]. Hence, no definitive conclusion could be made in favor or not of intensive lowering of serum uric acid levels in light of a putative higher risk for neurological diseases [103, 104]. Further mechanistic studies are needed in this field, using appropriate animal models for each distinct disease entity. Also, clinical trials of increasing serum uric acid in neurological disorders have been conducted [105, 106].

According to class I–IV evidence, physical activity was found to exert a beneficial effect against PD, AD, and all types/vascular dementia. Physical exercise can increase serum uric acid levels, which has been associated with a lower risk of developing PD and dementia [82, 104]. However, patients with PD may be unable to exercise much owing to neurological dysfunction, which might indicate reverse causation [107].

Serum vitamins B_12_, C, and D levels were associated with a lower risk of different neurological conditions, such as MS (as also reported recently [108]), AD, dementia/cognitive impairment, and PD. Around 80% of these meta-analyses represented heterogeneous evidence (*I*^2^ > 50%), which cautioned against false interpretations. The observed heterogeneity most likely arose from different comparison groups in prospective, retrospective, and case-control studies, causing some of the meta-analyses to be derived from studies with diverse, contrasted categories of serum vitamin B_12_, C, and D levels [49–51]. Furthermore, strong evidence links the presence of anti-EBV antibodies to MS (for further discussion, see [109]).

Our meta-umbrella review provides some evidence for a positive association of exposure to farming, pesticides, low-frequency electromagnetic fields, organic solvents, and *C. pneumonia* infection with the occurrence of several neurological conditions (such as MS, PD, and ALS). However, most of these associations were based on class III and IV evidence, which could have resulted from the substantial heterogeneity among the primary studies. Hence, these associations warrant cautious interpretation. We also suggest that the findings on chronic cerebrospinal venous insufficiency should be interpreted with caution, considering both the wide range of the corresponding confidence intervals and previous reports in the field [110, 111].

Framing our *meta-umbrella* review into the broader context of studies reviewing risk and protective factors for neurological disorders, we noticed that, in another comprehensive review of systematic reviews, for example, exposure to pesticides was identified as the commonest risk factor for AD, ALS, and PD, whereas smoking was associated with AD and MS [112].

### Smoking as an *exemplar* of studying risk and protective factors for neurological disorders

Below, we discuss the findings on the effects of tobacco smoking in a separate section. This choice was made because we consider that, with all the body of evidence surrounding this field, smoking should represent an exemplar for studying risk and protective factors for neurological disorders, or, as other authors have previously claimed, represents *the poster child of causal relations* [113]. We found that tobacco smoking is linked to an increased risk of MS (class I evidence), dementia (class IV evidence), and vascular dementia (class IV evidence), but also to a decreased risk of PD (class II evidence). A positive association exists between tobacco smoking and MS, with convincing (i.e., class I) evidence of, at least, a modest effect [51], even though confounding effects cannot be totally denied. More broadly, tobacco smoking has been included in the five principal risk factors that could explain around two out of three initial manifestations of demyelination (further reviewed in [114, 115]). Mechanistically, adverse immuno-modulatory effects, demyelination, and the disruption of the blood-brain barrier could be accountable for the positive association between smoking and MS, even though this remains to be proven [116]. Of note, the effects of smoking are now well-established regarding lung inflammation, the latter also linked to a high risk for MS [115]. Of particular interest is also the role of oral tobacco (*snuff*) usage, which was considered to be associated with a lower risk of MS, potentially through nicotine-mediated effects on subunits of immune cells expressing acetylcholine receptor [115].

Another possibility could be that people suffering from a certain neurological disorder, such as MS, prefer to smoke, whereas those unaffected choose to stop smoking more easily, as previously observed in patients with schizophrenia [117]. Therefore, there is concern that, since retrospective studies had been included in the initial meta-analyses, these could have introduced a bias in the relevant results of this *meta-umbrella* approach. Perhaps, in this specific field, it would have been probably wiser to select only the meta-analyses of prospective studies among the umbrella reviews. Similarly, another possibility could be to consider only umbrella reviews that have examined *credibility ceilings* [118], in order to assess effect estimates in combination with other sensitivity analyses (i.e., to include only prospective studies to assess temporality and reverse causation, or to perform the so-called *credibility ceilings*, which take into consideration limitations regarding the methodology of the studies) [37, 69, 119]. Nonetheless, this option would have been a rather laborious process in the context of this, already extensive, *meta-umbrella* approach. Besides, it is commonly known that extensively performing sub-analyses in many subgroups could be linked to artificially increasing events of statistical significance. In every case, the *teaching example* of cross-sectional studies on lung cancer and smoking (in which case, patients with lung cancer tend to quit smoking) for causing inverse causation should always be kept in mind [37].

With regard to PD, the potential underlying genetic and non-genetic roots (or/and bias) of the association between tobacco smoking and PD are reviewed elsewhere [120]. However, caution is needed in distinguishing epidemiological terminology (e.g., *suggesting that longer duration of smoking is needed for a risk reduction*, as cited in the above study) from core public health messages.

In every case, we feel that the core message of promoting tobacco smoking cessation as an effective public health intervention should remain undiluted because of its several well-established positive health effects [121, 122], irrespective of whether tobacco cessation might also decrease the incidence and/or severity of MS [123] and regardless of genetic susceptibility to smoking habits [121, 124]. Thus, we feel that the example of smoking, acting both as a risk factor for certain diseases and a protective factor for others, should not serve as an opportunity of potentially diluting a key public health message, or even counseling MS-affected patients or their family members who are at higher-than-normal risk in favor of smoking [125].

### Additional features with class I evidence

Below, we wish to highlight some additional features with class I evidence. Chronic occupational exposure to lead presented a higher risk for ALS. Arguably, lead toxicity represents a major underlying mechanism in ALS-related pathogenicity [126, 127]. In humans, lead toxicity manifests as clinical symptoms similar to those in ALS, such as weakness originating in the finger extensors or the wrist and, ultimately, spreading to additional muscles. Also, the blood of patients with ALS revealed higher levels of lead exposure-biomarkers than the respective levels in healthy controls [127]. In potential future research, lead toxicity should not be considered *in vacuum* but rather in association with other heavy metals and welding, even though the latter two are classified in lower levels of evidence (class II and class III, respectively). Moreover, when lead levels are taken into account, the association of another heavy metal, i.e., copper, with ALS risk becomes attenuated, suggesting a chief role of lead [128], even though certain isotopic compositions of copper have been detected at higher levels in the CSF of ALS patients than of AD patients or healthy controls in other studies [129]. Interestingly, occupational exposure to silica has also been implicated in ALS risk [130]; thus, it would be worth exploring whether silica (which belongs to the same family of the periodic table as lead does) could explain these traits.

Constipation was positively associated with PD (class 1 evidence). A prospective cohort study reported a significant association with a similar effect size in meta-analyses (reviewed in [50]). Another study reported that constipation could be a symptom of PD but also a premorbid symptom preceding motor dysfunction symptoms of PD by at least 10–20 years [131]. Nowadays, constipation is regarded as a manifestation of PD via the peripheral nervous system, a condition in which the threshold for the appearance of symptoms may be decreased. This is perhaps because of the larger functional reserve of the midbrain dopamine and integrated basal ganglia motor systems to control movement [132]. In any case, the connection between PD and gut dysfunction seems quite solid. In this context, laboratory studies have demonstrated an abnormal deposition of α-synuclein within the enteric nervous system, while, recently, the gut-to-brain α-synuclein’s spread (which is related to the Braak hypothesis) through the vagus nerve has been demonstrated in mouse models [50, 133, 134]. Moreover, a recent study of a huge cohort of 1.6 million subjects reported that the physiological human appendix contains intraneuronal α-synuclein and misfolded aggregates, and that removing the appendix early in life reduces the risk of developing PD [135]. Lastly, any causal association between beta-2-adrenoreceptor antagonist (beta-blocker) and higher risk for PD appears weak in terms of its evidence [136].

Our meta-umbrella review assessed specific risk factors related to dementia and AD. While only late-life depression and type 2 diabetes mellitus were positively associated with AD, depression at any stage in life was linked to all types of dementia. In fact, late-life depression was markedly associated with both dementia (vascular/all types) and AD [137]. It is still obscure whether depression is a risk factor for developing dementia or just a prodrome of dementia manifested by progressive cognitive decline [138]. The class II evidence of the association of type 2 diabetes mellitus with all types of dementia might reflect type 2 diabetes mellitus-driven susceptibility to different types of dementia, with a modest increase in the risk for AD [49].

Low levels of social interaction markedly affected the occurrence of dementia. Thus, social networking, along with educational and leisure activities, are modifiable protective factors, which might aid in the maintenance of cognitive function with increasing age (for systematic reviews of modifiable factors in dementia, see [139, 140]). The above could reflect the notion of *brain reserve*, which describes an individual’s ability to not develop the disease phenotype despite brain pathological changes that are either age- or disease-specific [49, 141].

Lastly, while serum 25-hydroxy-vitamin D has been investigated in umbrella reviews of neurological disorders, the same does not hold true for 1,25-hydroxy-vitamin D, as the latter has only been assessed in cancer [52].

Overall, despite this extensive body of evidence, we wish to emphasize that the majority of epidemiologically identified risk and protective factors do not lie at the bottom of the *health impact pyramid*, in which the main social and economic determinants of health, such as education, race, housing, and income, are included [142] (for an umbrella review on how these determinants affect health, see [143]). Interestingly, modification of these factors is expected to have the most pronounced impact at the population level, even though they have received significantly less research attention than socioeconomic determinants—an issue of health equity we have attempted to address elsewhere [124]. Thus, core public health actions should be undertaken not only top-down but also bottom-up, i.e., tackling not only the disease-specific but also the fundamental determinants of health [144–146].

In addition, there are potentially less appreciated or less easily quantifiable risk and protective factors, such as (a) the family environment (now-studied through *Family-Wide Association Studies* [147]); (b) the accumulation of physical and emotional stress along the human lifespan [148]; (c) living in urban versus rural environments, and in slum versus non-slum urban environments [149–151]; and (d) specific nutritional habits, such as milk and milk product consumption [152]. These factors may be worth exploring in the future, regarding their association with specific and integrated neurological conditions, thus combining *epidemiological and environmental neuroscience* [153]).

### Implications for target groups

Major implications for several target groups, namely patients and their caregivers, healthy subjects, clinicians, researchers, environmental health specialists, policy makers, and educational institutions, could be anticipated from this meta-umbrella review. In a way similar to umbrella reviews in other fields [34, 154], this *meta-umbrella* study provides the opportunity to (a) stimulate more comprehensive, patient-centered approaches, allowing truly informed decisions during genetic counseling or/and coaching for lifestyle changes [154–156]; (b) enhance the accuracy of predicted onset and natural history of neurological conditions at high-risk populations, especially if coupled with polygenic risk scores [157], and, in doing so, our study can help advocate disease prognostication based on the identified risk and protective factors; (c) offer guidance on future prevention interventions to mitigate amenable risk factors and promote protective factors in the general population, especially in young and middle-aged individuals, in whom the so-called *window of opportunity* still exists [158]; in that context, our approach could assist in promoting campaigns on brain health aimed towards the general public and could increase the level of awareness of neurological conditions, following the successful examples of campaigns regarding cancer and cardiovascular conditions; (d) assist policy makers at the local, national, regional, international, and global level to draft new guidelines or update existing ones, and to explore how modifying risk and protective factors should be incorporated into national health plans; (e) stimulate additional mechanistic, translational, and clinical research on the etiology of neurological conditions and the many unanswered questions; and equally importantly, (f) assess the associations between several risk and protective factors and specific neurological conditions in terms of their natural history and magnitude, which represents a gap in the literature; (g) generate a broader discussion on the role of umbrella and *meta-umbrella* review approaches as the highest level of evidence in the *meta-research* field; (h) serve as teaching material for courses on preventive neurology offered by the relevant medical education institutions; (i) contribute to helping physicians understand the contribution of *environmental elements* as risk factors for neurological disorders, to assist environmental health specialists in the appreciation of the ties between the nervous system and environmental health; and (k) to address these factors (i.e., mitigate the risk factors and enhance the protective factors) by taking action starting from early and middle adulthood, thus ultimately reducing to some extent the incidence and, hence, the prevalence of neurological disorders.

Although others have argued that deciphering how the mechanistic effects of certain risk and protective factors are different between distinct neurological disorders (e.g., AD and ALS) [159], we feel that maintaining a public health lens approach is always crucial. In this case, the commonality of some risk and protective factors could present an opportunity for *holistic* policy making (e.g., promoting Mediterranean diet to prevent a wide spectrum of neurological disorders), and it could also serve as an impetus in developing *transdiagnostic* approaches in neurology, similarly to psychiatry [34] (for further discussion on the *transdiagnostic theory*, see [160, 161]).

In addition, our approach would advocate the development of appropriate statistical tools to account for the fractions of affected neurological populations vs. risk and protective factors, in alignment with previous approaches (e.g., [162]). In the latter context, this study may lead to developing criteria and tools that are essential in the investigation of the quality of umbrella reviews, allowing inter-comparisons between such analyses. In the same direction, there is a need for consistent a priori *publication of protocol* for umbrella reviews, in alignment with previous calls [163]. Further adherence to common, standardized methodologies could be improved in accordance with previous suggestions (as commented in [54]).

Many of the class I evidence results in our study re-affirm previous opinions on implementation science (for a discussion on geopolitical factors affecting implementation of policies on chronic diseases, see [10]). As previously supported [164], *scientists should stop advocating the need for yet another clinical trial on the cognitive benefits of healthy lifestyles and lobby decision-makers to implement societal polices to actively promote propitious lifestyles. This approach will substantially produce benefit not only for the brain but for the society at large*. Complicated situations in which a factor has both a beneficial and a risk effect (e.g., hypertension in PD and dementia) provide an opportunity to highlight the broader potential discrepancies between public health and precision medicine [124]. Interestingly, this gap in the research literature also calls for *implementation science research* to guide health policy and to be a major component of the so-called *population health science* [32].

### Strengths of our *meta-umbrella* approach

This study has several strengths, as it is public health policy-, clinical science-, and *meta-research*-oriented; thus, it represents a call-for-action, similar to similar calls in other diseases [165]. The first strength includes the use of a methodical and systematic approach in gathering and evaluating all published, appropriate-quality umbrella literature regarding protective and risk factors for the chronic non-communicable neurological disorders. This may be quite useful to the busy clinician who may not have adequate time to perform reviews on his/her own [166], and who, in turn, is offered an *overarching* and up-to-date knowledge on a wide array of contributing epidemiological factors. In this context, our approach attempts to address the challenge of evaluating evidence provided by a number of high-quality meta-analyses and, in turn, umbrella reviews [167].

Our systematic, *overarching* approach allows studying the top *evidence*. In general, umbrella designs are of special value when applied to provide an overall picture that can inform guidelines. For instance, rather than examining one risk factor or disease, a meta-umbrella review can consider multiple risk factors for multiple disease processes. For this reason, we ensured that four researchers participated in the search and quality assessment of published studies, thus, enhancing the validity of our analyses. Notably, whereas earlier non-umbrella reviews examined 14 neurological disorders (and the associated risk and protective factors) as part of National Health Guidelines [63], our study attempted to review all neurological disorders in the sum of relevant umbrella reviews. This approach becomes more important if we consider that umbrella reviews themselves are already regarded as the highest level of evidence in the hierarchical pyramid.

In the same context, by performing an umbrella review, a neurology-wide notion is created based on the extent to which systematic reviews produce similar results and conclusions and, in turn, unravel the consistency or contradiction of evidence in this field (as commented in [2]). In this sense, by co-examining several factors in parallel, umbrella reviews, and, as a corollary, our *meta-umbrella* approach, can detect irregular patterns in the associations observed (e.g., as the case for farming and pesticides in [167]). To enhance the encompassing character of our study, we included biomarkers acting as surrogate measurements of epidemiological or/and clinical features. Although these surrogates might lower the strength of the evidence, they can still be indicative of the above features and, thus, offer potentially valuable observations.

Our meta-umbrella approach should be viewed as a *third-generation systematic review* study design, allowing, among others, to discuss research gaps and to determine the potential mechanistic underpinnings of such associations. In other words, because of the advent in the wealth of umbrella reviews conducted, this *meta-umbrella* review should be seen as a *logical next step*, where available umbrella reviews will serve as the *analytical unit of the review*, in which the *meta-umbrella* approach will allow selecting and including the *highest level of evidence*, *notably other umbrella reviews* (i.e., being the *meta* equivalent of umbrella reviews, following their description in [2]).

This study obviates the need to perform further research on many different factors demonstrating no significant association, as shown in previous studies [125]. In that context, it is important to cautiously interpret *p* values and CIs. Indeed, when a *p* value is within the non-significant range, this means that either the groups have no difference or, alternatively, that the participants’ number is too low to show if such a difference exists. Similarly, when a confidence interval lies in both sides of the line of no effect, this means that either the groups have no difference, or, alternatively, that the number of participants (sample size) is too small to show if such a difference exists. Conversely, a confidence interval not crossing the line of no effect can provide reassurance on the strength or weakness of the evidence, and importantly, whether the study’s results are definitive, with no further need to perform additional studies on the particular issue. Therefore, these notions should be considered while interpreting the results of this meta-umbrella study.

### Limitations of our *meta-umbrella* approach

Some limitations should be acknowledged when considering this meta-umbrella study. First, the population and interventions discussed could be perceived as too broad. Our study, however, was in alignment with its *overarching* goal. Also, the wide presentation of risk and protective factors could be perceived as attenuated if not followed by investigations on their biological plausibility, thus calling for studies to bridge the gap between epidemiology and pathobiology of neurological diseases.

Second, our analyses of the existing umbrella reviews heavily relied both on the quality of information and on the meta-analytical methods employed by the included umbrella reviews, an issue that may have some major caveats. Indeed, all negative aspects from the original studies, which may harbor distinct study designs, classification criteria, and sample sizes that should preferably not be combined in meta-analyses, can be *transferred* to the meta-analyses, umbrella reviews, and herein, our *meta-umbrella* review (discussed further in [78]). For example, it has been suggested that, in the case of only a few studies (i.e., with less than one thousand patients) examining a specific risk or protective factor, which may otherwise have a very strong effect, this factor may still be classified as class IV evidence [37]. This is because, by the very nature of the study design, the quality of evidence offered by (and the conclusions derived from) this *meta-umbrella* approach cannot surpass that of the umbrella reviews [125]. Likewise, the biases and limitations of the meta-analyses, including those relevant to confounding factors, are *transferred* to the umbrella and meta-umbrella reviews [37, 54]. Collectively, our *meta-umbrella* review depends on the choices of the umbrella systematic reviewers, putting confidence on their choices [154].

A third caveat is that a risk or protective factor, or even systematic reviews not previously analyzed in umbrella reviews, may be neglected in a *meta-umbrella* study. For instance, the role of physical and emotional stress is not discussed in (or even captured) umbrella reviews, while the same holds true for pregnancy-related maternal health, epilepsy, myasthenia, Tourette syndrome, and Huntington disease, to name a few examples (in contrast to other kind of reviews examining these factors [112]). Likewise, certain factors, such as air pollution and other environmental risks, appear under-investigated by the umbrella reviews examined, despite their major contribution to the pathophysiology of chronic diseases [168]. Similarly, *intermediate* risk factors (as defined in [169]), such as birth weight, or other increasingly recognized factors, such as social networks, the impact of which on health outcomes is being steadily better understood [170], could have been neglected by umbrella reviews. Another major example is that studies on early life risk factors, including in utero exposure, seem absent in umbrella reviews, even though the early life-related factors (and even those evoked in a transgenerational manner by progenies, e.g., through epigenetic regulations [171–173], or those described in the capacity-load model [174]) play crucial roles in the development of adult diseases [56, 57, 175]. Interpreting these studies with caution is essential as, at least in theory, several early life factors may influence the intercept of adult disease manifestations, whereas other factors may exercise an influence later in the disease course. The above limitation could be studied in detail in the future.

That said, with regard to the majority of risk and protective factors, which cannot be examined using standard epidemiological approaches or are not frequently assessed, the level of evidence pertaining to these factors will be most likely low given the scarcity of the existing relevant data (as further discussed in [34]). Similarly, in light of the massive amount of primary and secondary research studies, the possibility of a medical field being totally unexplored at the systematic review/meta-analysis level is not high [37].

Umbrella reviews and, as corollary, our *meta-umbrella* approach, are inherently biased in favor of commonly assessed factors, towards more common neurological disorders (perhaps with the exception of neuromyelitis optica), or towards those of adult but not pediatric populations, because, so far, all umbrella reviews have been conducted on these disorders. This is because of the lack of umbrella reviews examining factors that are less common in rare or adult neurological disorders, besides the conditions included. Assessing the input of genetic and environmental factors in rare diseases by comparing evidence from common diseases could be hampered by the heterogeneity of studies (further discussed in [78]). Perhaps, key events may lie in the interactions of genetic and non-purely genetic factors, such as epigenetic modifications (e.g., DNA methylation, chromatin modifications, etc.), the gut microbiome, and others, summing up to the so-called *exposome*—a field that is still in its infancy [176]. Likewise, our study may be biased towards more easily quantifiable factors, neglecting other crucial factors, such as stress—a factor established nowadays for its multiple negative health effects, including neurological disorders [177–181].

Despite our efforts to be as thorough as possible in employing a sensitive literature search strategy, no umbrella reviews except in the English language were found in our systematic literature search. However, we cannot exclude the likelihood that some relevant papers in other languages might have been overlooked, even though Moher et al. have shown that omitting studies in non-English languages may not introduce considerable bias [182]. Similarly, while we assessed the main results of the reviews, we may have overlooked the unreported protective and risk factors that could have been significant. Likewise, some existing evidence might have been omitted in our meta-umbrella review because some studies might have been omitted or were not included in the prior meta-analyses or, because of the blossoming field of umbrella reviews, some studies might have been published after September 21, 2018. To this end, and following the example of previous studies [89], we also performed an additional search for all relevant publications (umbrella reviews) that have appeared in PubMed until January 1, 2020, so that the reader remains updated (Additional file 3) [46, 83, 84, 183–202]. Moreover, despite our search strategy to include gray literature and policy documents, following previous urging [2], no relevant documents were identified.

It is possible that conclusions drawn in some studies may have been confounded by biases other than sample size or by residual confounding effects per se [203]. For example, reverse causality might operate in the associations assessed, and it might have affected the findings regarding the nature of linkage. This consideration highlights the need for prospective studies to demonstrate the direction of causality, and the nature of linkage. This issue becomes important when a broad range of disorders are considered (e.g., association of smoking with stroke vs. that with lung cancer metastases to the brain). Similar considerations could also include other sources of bias, either unknown or known, e.g., the so-called survivor bias (as in ALS, or stroke). This latter bias refers to factors that are highly prevalent following the disease onset; these factors can turn out to be protective. The phenomenon is worse in studies assessing the prevalence of a disease, because factors contributing to both the disease’s onset and its progression may be underestimated (as reviewed in [159]). Moreover, several additional types of bias remain unaddressed. These could include sex bias, how controls are selected, how the measures of exposure are defined, and the composition of the population examined in terms of demographic and geographical variation (for further discussion on these issues, see [125]). More broadly, causal inference vs. investigation of mere *correlates* nowadays requires sophisticated methods (e.g., multivariable Mendelian randomization or Bayesian approaches), the description and usage of which exceed the aims of the present study (for a discussion, see [113, 204]).

Another source of bias could refer to the heterogeneity of the data in combining risk and protective factors for multiple different disease processes. Multiple risk factors, which may be ill-defined or based on studies that are observational in design, may be associated with very distinct disease processes, with differing pathophysiologies. For example, PD is particularly prone to recall bias as it tends to have a longer prodromal phase with symptoms that might not be diagnosed as PD until manifestation of the later characteristic motor symptoms. Likewise, the comparator groups for different neurological disorders included in this meta-umbrella review are expected to be different. Importantly, the so-called *vibration of effects* in observational studies, which is linked to how the selection of adjusting variables leads to results’ variability, should be considered while assessing our results [205].

The *overarching* character of presenting more-than-one protective and risk factors for the sum of neurological conditions should be regarded through the *public health* and *clinical counseling lenses* rather than from a mere *epidemiological* perspective. One of our chief goals was to reduce reporting bias—this is, indeed, one of the main reasons for which systematic reviews (in our case a systematic review of umbrella reviews) are performed. In doing so, we wish to highlight (a) the challenges preventive medicine specialists can face (and the need for balanced, informed decisions outweighing benefits vs. harms in both societal and personalized approaches) during counseling services, especially with regard to how well they can communicate their message, e.g., when the same factor is protective for one neurological disease and risk factor for the other, and (b) the potential inherent difficulties of forming public health guidelines.

In the same context, other limitations could include (a) the potentially different, or, in some cases, potentially inaccurate definitions regarding healthy control groups among meta-analyses or/and individual studies leading to inaccuracies, thus introducing another source of bias (commented in ([34]); and (b) the fact that, in some cases, the protective and risk factors examined may be due to the interaction of other, more *primordial* factors (e.g., educational level as result of parental education and family wealth status), therefore representing risk markers, or that there is an apparent heterogeneity in the definition of these factors [54]. That said, and in alignment with previous studies [34], we pursued a *pragmatic approach* by showing confidence to the choice of the primary systematic reviewers—and, this should be recognized [37].

Of note, the possibility of non-uniform diagnostic criteria for the disorders used for study selection in the primary meta-analyses gathered by the umbrella reviews is also a limitation that should be considered when comparing different factors for a particular disorder, especially in the context of umbrella reviews assessing a single factor vs. several disorders; this is particularly important for disorders such as AD and cognitive impairment. For instance, some umbrella reviews might restrict pooling to studies that have used only particular criteria, even though the original meta-analysis might have included more studies with more permissive criteria (such as “assessment by attending doctor”). Ideally, extracting detailed information from the primary meta-analyses (or the summary table with meta-analysis characteristics available in some umbrella reviews) would be needed, as it is difficult to assess these particularities at the level of the meta-umbrella review; however, such a task could not be easily performed in our study because (a) our initially designed pragmatic approach followed the definition of the umbrella reviews, (b) there were no informaticians at our library facilities to assist in this task, (c) umbrella reviews, in most cases, do not include the inclusion criteria of the original studies, and (d) if we had decided to set out for the above task, then we would have had to list the inclusion criteria of all the primary studies, which were presumably different.

Moreover, the issue of overlapping systematic reviews cannot be excluded, although we have undertaken every effort to retain systematic reviews and meta-analyses that included the highest number of primary studies. Therefore, as discussed elsewhere [167], similar direction (i.e., positive or negative association) and order of magnitude may be due to similar biases existing in these overlapping studies. It would have been, nevertheless, preferable if we had created a data matrix previously described as *Corrected Covered Area*, to assess overlapping (for further description, see [42]).

Of note, our approach presents additional difficulties in comparing the effect sizes spanning the sum of factors investigated, due to the issue of directly comparing the effect sizes between different meta-analyses (e.g., HR, RR, mean difference, and standardized mean difference), let alone when these effect sizes stemmed from different study designs (e.g., HR presents difficulties if used and interpreted in cross-sectional studies) or when distinct effect size metrics are not converted to a single metric of reference. Thus, our study’s results should be interpreted more through a qualitative rather than a quantitative lens [37]. Additionally, pooled effects cannot be estimated—the same is pertinent to umbrella reviews of meta-analyses. In this context, data could have been presented in forest plots without depicting pooled effects [37]. Furthermore, another effect size, the attributable risk (AR), has the potential of assessing public health impact, because it allows capturing how much an outcome could have been prevented from occurring if there was no exposure to a certain factor [206]. Nevertheless, AR has not been frequently used in systematic reviews [89]. Thus, future studies should be encouraged to focus on calculating the AR for neurological disorders based on the exposure to all risk and protective factors, to ultimately guide health and public policy interventions. In addition, previous studies have suggested replacing the term *risk factor* with *predictor* and *explanatory factor* for risk stratification and causal studies, respectively [70]. Similarly, others have proposed the notion of *risk markers* to reflect that many risk factors, such as immigration and ethnicity, may stem from the interaction of other risk factors [34]. Collectively, future umbrella and *meta-umbrella* studies should agree on the effect size to be used for comparisons and on the preferred terminology regarding risk and protective factors (for the lack of standardization in medical terminology, see [207]). In the same context, an additional consideration could be that, as shown in Table 4, most umbrella reviews discussed herein applied their own definition regarding the credibility of epidemiological evidence, consequentially hindering inter-comparisons and, thus, calling for a uniform set of criteria in future umbrella reviews.

**Table 4:** Level of evidence in various studies

Level	I / Convincing	II / Highly suggestive / Probable	III / Suggestive / Possible	IV / Weak / Limited-contrasting
Grosso, 2017 [76]	High: concordance between meta-analyses of RCTs and meta-analyses of observational studies; low: meta-analyses of RCTs with results contrary to those from meta-analyses of observational studies	High: meta-analyses of prospective studies with no heterogeneity, no potential confounding factors identified, and agreement of results over time and among meta-analyses, including studies with different designs; medium: meta-analyses of prospective studies with no heterogeneity and no potential confounding factors identified; low: meta-analyses of prospective and case-control studies with no heterogeneity and no potential confounding factors identified	High: meta-analyses of prospective studies lacking information on heterogeneity and potential confounding factors; medium: meta-analyses of prospective and case-control studies lacking information on heterogeneity and potential confounding factors; low: meta-analyses of case-control studies or meta-analyses of any other study design with significant heterogeneity (*I*^2^ > 50%) and potential confounding factors	Limited studies included in meta-analyses (*n* ≤ 3) or evident contrasting results from meta-analyses with the same level of evidence
Veronese, 2018 [77] Veronese, 2019 [83] Li, 2017 [86]	Statistical significance with *p* < 10^− 6^, more than 1000 cases (or > 20,000 participants for continuous outcomes), the largest component study reported statistically significant effect (*p* < 0.05); 95% PI excluded the null; no large heterogeneity (*I*^2^ < 50%), no evidence of small-study effects (*p* > 0.10) and excess significance bias (*p* > 0.10)	Statistical significance with *p* < 10^− 6^, more than 1000 cases (or > 20,000 participants for continuous outcomes), the largest component study reported statistically significant effect (*p* < 0.05)	Statistical significance with *p* < 10^− 3^, more than 1000 cases (or > 20,000 participants for continuous outcomes)	The remaining statistically significant associations with *p* < 0.05.
Dinu, 2018 [81]	Significance threshold reached at *p* ≤ 0.001 for both random and fixed effects calculation; > 1000 cases (or > 5000 total participants if the metric was continuous); not large heterogeneity between studies (*I*^2^ < 50%); 95% PI excluding the null value; no evidence of small-study effects (if it could be tested)	Significance threshold reached at *p* ≤ 0.001 for both random and fixed effects calculation; > 1000 cases (or > 5000 total participants if the metric was continuous); not considerable heterogeneity between studies (*I*^2^ = 50–75%)	Significance threshold reached at *p* ≤ 0.001 for random effect calculation; 500–1000 cases (or 2500–5000 total participants if the metric was continuous)	Significance threshold reached at *p* ≤ 0.05 for random effects calculation
Theodoratou, 2014 [52]	Evidence existed from both observational studies and RCTs, and association/effect was of the same direction, statistically significant at *p* ≤ 0.001, and free from bias	Evidence existed from both observational studies and RCTs, and association/effect was of the same direction and statistically significant at *p* ≤ 0.001, but excess significance could not be tested; or evidence existed from RCTs and effect was statistically significant at *p* ≤ 0.001 and with no contrary results from observational data (that is, systematic reviews, if any exist, are also definitive or suggestive and meta-analyses of observational studies, if any exist, are in the same direction)	Suggestive: Evidence from RCTs with an effect at 0.001 ≤ *p* ≤ 0.05 and with no contrary results from observational data (same as above); or evidence from meta-analyses of observational studies showing an association at *p* ≤ 0.001, with no contrary results from randomized data (that is, meta-analysis of RCTs, if present, have effects in the same direction) and, if it could be tested, no evidence of small-study effects (*p* ≥ 0.10), not very large heterogeneity (*I*^2^ ≤ 75%), no evidence for excess significance, based on cumulative evidence of more than 500 disease events (or more than 5000 total participants if type of metric was continuous)	[Substantial effect unlikely]: Evidence from observational studies or RCTs enough to conclude that a substantial effect is unlikely based on the magnitude and the significance level
Belbasis, 2016 [78] Belbasis, 2015 [18, 51]	More than 1000 cases, significant summary associations (*p* < 0.001) per random effects calculations, no evidence of small-study effects, no evidence for excess significance bias, PI not including the null, and not large heterogeneity (*I*^2^ ≤ 50%)effects and excess significance	[No such category exists in these studies]	Nominally significant summary associations (*p* < 0.05) per random effects calculations, no evidence of small-study effects, no evidence for excess significance bias, and not large heterogeneity (*I*^2^ < 50%)	All other risk factors with nominally significant summary associations (*p* < 0.05);
Bellou, 2017 [49] Bellou, 2016 [50]	The associations that fulfilled all the following criteria: statistical significance according to random effects model at *p* < 10^− 6^; based on more than 1000 cases; without large between-study heterogeneity (*I*^2^ < 50%); 95% PI excluding the null value; and no evidence of small-study effects and excess significance	Associations with > 1000 cases, *p* < 10^− 6^, and largest study presenting a statistically significant effect (with 95% CI excluding the null value)	The associations supported by > 1000 cases and a significant effect at *p* < 10^− 3^)	All other risk factors with nominally significant summary associations (*p* < 0.05)
Poole, 2017 [79]	The classification was based on AMSTAR (A Measurement tool to Assess Systematic Reviews), as following: Q1: A-priori design; Q2: Duplicate study selection and data extraction; Q3: Search comprehensiveness; Q4: Inclusion of gray literature; Q5: Included and excluded studies provided; Q6: Characteristics of the included studies provided; Q7: Scientific quality of the primary studies assessed and documented; Q8: Scientific quality of included studies used appropriately in formulating conclusions; Q9: Appropriateness of methods used to combine studies’ findings; Q10: Likelihood of publication bias was assessed; Q11: Conflict of interest-potential sources of support were clearly acknowledged in both the systematic review and the included studies.			
McRae, 2017 [85] Galbete, 2018 [84] Posadzki, 2018 [73]	Not available levels of evidence (*)			

*Abbreviations*: *RCT* randomized controlled trials, *PI* prediction interval, *95% CI* 95% confidence interval

*Non-pragmatic approach was applied in this meta-umbrella review

Another limitation is that our *meta-umbrella* review was not registered for its goal or protocol at a database like PROSPERO, although (a) our search criteria were predefined and typical of systematic reviews, (b) our exclusion criteria were not intending to alter the number or nature of umbrella reviews identified and (c) no *re-synthesis* or quantitative synthesis with regard to outcomes of interest or of any other data in any sort of meta-analysis (as discussed in [2]) took place. Besides, there has been no established protocol to assess the quality of umbrella reviews; thus, AMSTAR application should be treated with caution.

Other limitations could be that (a) we merged the systematic reviews referring to observational studies with those of clinical trials, given that observational studies should be treated with high cautiousness when referring to risk and protective factors (commented in [208]); (b) we did not distinguish between the two major categories of observational studies, i.e., case-control vs. cohort studies; and (c) we did not perform a sensitivity analysis. Recently, interesting methodologies have been proposed to reconcile the distinct study designs of observational studies and randomized trials [209]. Nonetheless, we feel that the above *merging* of distinct study design types, which holds an undeniable bias in principle, has not impacted our results, because of (a) our *pragmatic* approach, (b) the non-quantitative approach applied herein, and (c) the lack of evident *internal* discrepancy among the findings reviewed. Besides, both study types offer *real-word results* (commented in [167]). Another similar concern could be why retrospective studies were included in this *meta-umbrella* review, especially for those influential factors that cannot be randomized (smoking). Nevertheless, selecting for retrospective studies to include can affect, in principle, the meta-analyses (e.g., there are meta-analyses of observational or cohort studies) and not a meta-umbrella review. In this sense, performing a sensitivity analysis may not be feasible for a *meta-umbrella* review. In addition, the fact that umbrella reviews only use statistical testing to show the existence of bias and cannot provide evidence of their nature and extent should be taken into account, alongside with the fact that umbrella reviews cannot supply any comparative ranking, as is done in a network meta-analysis.

Lastly, our search strategy for umbrella reviews did not allow identification of studies titled *systematic reviews of systematic reviews*, *meta-reviews*, *systematic meta-reviews* (examples of this *study types include* [210–212]), *systematic reviews of meta-analyses, meta-epidemiological studies, overviews, field-wide meta-analyses, series of systematic reviews and meta-analyses, synthesis of systematic reviews, meta-meta-analyses, research-on-research studies, comprehensive reviews, reviews of meta-analyses*, or *combinations* of the above (examples in [42, 213–216]). However, omitting such studies does not negate our primary goal to consider only the data reported in reviews clearly defined as umbrella reviews. Besides, many of these types of studies, such as *overviews*, do not clearly describe systematic reviews, let alone in a homogeneous manner [97].

Moreover, an a posteriori search strategy using similar search strings revealed only one study with potential applicability (i.e., [217]). Under all circumstances, a call for uniform terminology in the field of *meta-research* could be made, in alignment with previously expressed opinions [207], in particular those arguing that the definitions of systematic reviews is surrounded by ambiguity and lack of clarity [218].

## Conclusions

Notwithstanding potential considerations that the topic under study may appear broad, this exposure-wide assessment of risk and protective factors of chronic neurological disorders, conducted using a systematic review of umbrella reviews (and the corresponding meta-analyses), offers an *overarching* outline of different aspects of the assumed risk and protective factors in the field. Such comprehensive approaches provide a way to systematically analyze the qualitative and quantitative characteristics of the existing literature, the validity of evidence, and how these studies could provide readers with information on knowledge gaps. In doing so, this *meta-umbrella* review allows to identify the sum, so far, of preventable or/and modifiable risk factors, which may be relevant—either as common to most or specific to certain—neurological disorders. To all these goals, standardizing how a study is designed and how exposure to certain factors is defined would be essential [125]. Likewise, we should be prompted to develop and standardize the criteria for assessing the quality of umbrella reviews.

In addition, our *meta-umbrella* approach provides a wealth of discussion on potential biases that could occur in reporting umbrella reviews, and it provides a rationale to develop reproducible robust methodologies while performing umbrella studies. To enhance extensive analyses of risk/protective factors, we recommend developing standardized and reproducible methods to identify these factors—performing a field-based systematic review of umbrella reviews might simplify the process. Equally importantly, as almost all umbrella review studies have so far focused on neurological disorders of resource-rich countries, a call for intensifying meta-research on neurological disorders affecting resource-poor countries could be addressed.

Lastly, this *meta-umbrella* review offers new perspectives in the meta-research field. The fact that not all risk factors or/and diseases were uniformly mentioned could per se serve as a call-for-action to develop guidelines and a uniform framework for *Systematic reviews of Umbrella reviews*. In the same context, our meta-umbrella review could serve as a call-for-action to conduct umbrella reviews using more harmonized criteria of evidence and strength of evidence; this could be performed by grouping studies by the criteria used to assess evidence, and then reaching conclusions in this regard.

Being distant from any overly expectations on reaching final conclusions on causal vs. casual associations, this study attempted to highlight the commonality of certain risk and protective factors, and the relevant level of evidence surrounding these factors [167]. Emphasis should also be given on (a) the challenges that preventive medicine specialists can face when offering counseling services and (b) the need to communicate health promotion messages to the patients’ community in a consistent manner. Interestingly, regarding the commonality of diseases in terms of symptoms, there seems to exist an underlying basis on shared genetic traits, as well as the level of connectivity in protein interactions (*disease network neighborhoods*) [219, 220]; however, in parallel to a public health approach, future additional research is essential to better understand the distinct pathophysiology of conditions affecting the nervous system. For example, a key question would be whether the cellular reactions following exposure to risk factors are genetically determined, and if so, whether this genetic background is similar between distinct neurological patients.

Under all circumstances, we feel that to be able to reap further epidemiological rewards, this sort of study (i.e., *meta-umbrella* review) should be jointly assessed with studies coupling mechanistic and biological insights with statistical methods on causal inference [113, 167]. Future research on the field may require a more thorough application of statistics focusing on causal effects, such as mediation (a statistical technique allowing to study the effects of a third *mediator* variable between an independent and dependent variable) and multivariable Mendelian randomization studies, which can render some risk factors as causal determinants [113]. However, caution on appropriately interpreting such methodologies has been expressed [221]. In parallel to this, novel solid evidence from novel umbrella reviews should steadily update this type of study (in a way similar to *living systematic review* [222]), which, in turn, could offer periodic updates for policy formulation (for an example on MS, see [223]). In doing so, we propose that “living” systematic reviews, defined as systematic reviews the content of which is updated in a continuous manner by including the most up-to-date evidence once the latter is available [224], should be expanded beyond their current focus on accelerated research areas.

Ultimately, neurological research and health policy trends could focus on creating a *Neurological Diseases Atlas*, in which every risk and protective factor will be both qualitatively and quantitively linked to all associated diseases (similarity to *causal mapping* [113]). This *Atlas* could be accompanied by a score-based approach we wish to call *poly-non-genic risk scores*, which could assess the exposure to common factors (in a similar pattern to polygenic scores). In doing so, this *Atlas*—if appropriately assessing mean percentage population attributable fractions of each risk or protective factor for the sum of diseases (as in [162], for a single disease)—can contribute to major clinical and public health impact.

## Supplementary Information

**Additional file 1.** PRISMA checklist.

**Additional file 2.** Appendices and Tables S1-S2. Appendix 1-Search strategy and results. Appendix 2-WHO definition of neurological disorders. Table S1-Qualitative and quantitative characteristics of the 203 eligible non-overlapping meta-analyses of non-purely genetic (environmental) risk and protective factors for chronic neurological diseases. Table S2-Methodological quality of included umbrella reviews based on the AMSTAR criteria and score.

**Additional file 3.** Updated search of umbrella reviews.

## Data Availability

Data sharing is not applicable to this article as no datasets were generated or analyzed during the current study.

## Abbreviations

AD: Alzheimer disease
AMSTAR: A Measurement Tool to Assess Systematic Reviews
ALS: Amyotrophic lateral sclerosis
AR: Attributable risk
BMI: Body mass index
CI: Confidence interval
CSF: Cerebrospinal fluid
EBNA: Epstein Barr nuclear antigen
EBV: Anti-Epstein-Barr virus
GWAS: Genome-wide association studies
IQR: Interquartile range
MS: Multiple sclerosis
NSAIDs: Nonsteroidal anti-inflammatory drugs
OR: Odds ratio
PD: Parkinson disease
PRISMA: Preferred Reporting Items for Systematic Reviews and Meta-analyses
RR: Risk ratio
SNPs: Single-nucleotide polymorphisms
