# Correction to: Non-genetic risk and protective factors and biomarkers for neurological disorders: a meta-umbrella systematic review of umbrella reviews

**DOI:** 10.1186/s12916-021-02159-2

**Published:** 2021-11-09

**Authors:** Alexios-Fotios A. Mentis, Efthimios Dardiotis, Vasiliki Efthymiou, George P. Chrousos

**Affiliations:** 1 Public Health Laboratories, Hellenic Pasteur Institute, Athens, Greece; 2 Department of Neurology, University Hospital of Larissa, University of Thessaly, Larissa, Greece; 3 University Research Institute of Maternal and Child Health and Precision Medicine, National and Kapodistrian University of Athens, Athens, Greece; 4 University Research Institute of Maternal and Child Health and Precision Medicine, and UNESCO Chair on Adolescent Health Care, National and Kapodistrian University of Athens, Athens, Greece

**Correction to: BMC Med 19, 6 (2021)**

**https://doi.org/10.1186/s12916-020-01873-7**

It was highlighted that the original article [1]contained an error under the section *Principal findings* specifically on page 12, the sentence “This systematic review of umbrella reviews revealed counterintuitively a significant association of low serum uric acid levels with a decreased risk of several neurological diseases” should read: “This systematic review of umbrella reviews revealed counterintuitely a significant association of high serum uric acid levels with a decreased risk of several neurological diseases”. The original article has been updated.
