# Supplementary material for: Non-genetic risk and protective factors and biomarkers for neurological disorders: a meta-umbrella systematic review of umbrella reviews
Source: BMC Med. 2021 Jan 13;19:6. doi: 10.1186/s12916-020-01873-7 (PMC7805241; doi:10.1186/s12916-020-01873-7)
Supplement: Supplementary file 2 — Additional file 2. Appendices and Tables S1-S2. Appendix 1-Search strategy and results. Appendix 2-WHO definition of neurological disorders. Table S1-Qualitative and quantitative characteristics of the 203 eligible non-overlapping meta-analyses of non-purely genetic (environmental) risk and protective factors for chronic neurological diseases. Table S2-Methodological quality of included umbrella reviews based on the AMSTAR criteria and score. [file 12916_2020_1873_MOESM2_ESM.docx]

**Appendix 1.**

- 1. **Search results**

| **Database** | **Hits** | **Relevant** |
| --- | --- | --- |
| PUBMED/MEDLINE | 1,178 | 21 |
| SCOPUS | 1,023 | 6 |
| Web of Science | 165 | 17 |
| CINAHL | 106 | 0 |
| Cochrane Database of Systematic Reviews | 17 | 0 |
| JBI Database of Systematic Reviews and Implementation Reports | 84 | 1 |
| DARE | 31 | 0 |
| PROSPERO register | 193 | 5 |
| ProQuest Dissertations & Theses | 0 | 0 |
| **Total** | **2,797** | **50** |

**1.2 Generic version of search strategy**

|  | **Search terms used alone or in combination** |
| --- | --- |
| 1 | (exp) (umbrella review$ OR umbrella review$.ti,ab.) |
| 2 | Multiple outcomes |
| 3 | stroke*, Alzheimer disease or dementia*, multiple sclerosis*, headache*, amyotrophic lateral sclerosis*, Parkinson disease, neurolog$ OR (multiple outcomes) |

**Appendix 2. WHO definition of neurological disorders**

The WHO definition of neurological disorders is as per the following excerpt:

*Neurological Disorders: Public Health Challenges*

*There is ample evidence that pinpoints neurological disorders as one of the greatest threats to public health. There are several gaps in understanding the many issues related to neurological disorders, but we already know enough about their nature and treatment to be able to shape effective policy responses to some of the most prevalent among them.*

*Neurological disorders: public health challenges describes and discusses the increasing global public health importance of common neurological disorders such as dementia, epilepsy, headache disorders, multiple sclerosis, neuroinfections, neurological disorders associated with malnutrition, pain associated with neurological disorders, Parkinson disease, stroke and traumatic brain injuries.*

*The book provides the public health perspective for these disorders and presents fresh and updated estimates and predictions of the global burden borne by them. It provides information and advice on public health interventions that may be applied to reduce the occurrence and consequences of neurological disorders. It offers health professionals and planners the opportunity to assess the burden caused by these disorders in their country and to take appropriate action.*

**Table S1.** Qualitative and quantitative characteristics of the 203 eligible non-overlapping meta-analyses of non-purely genetic (environmental) risk and protective factors for chronic neurological diseases (Table 1.a: Specific risk and protective factors versus different neurological diseases ─ 75 non-overlapping studies; Table 1.b.: Specific neurological diseases versus different protective and risk factors ─ 128 non-overlapping studies)

| **Table S1.a. Specific risk and protective factors versus different neurological diseases.** | | | | | | | | | | | | | | | | | | | | | | | |
| --- | --- | --- | --- | --- | --- | --- | --- | --- | --- | --- | --- | --- | --- | --- | --- | --- | --- | --- | --- | --- | --- | --- | --- |
| **Reference** | **Neurological disorder** | | **Level of comparison** | | **Total number of participants in each meta-analysis** | | **Number of primary studies** | | **Effect size metric** | | **Random-effects summary effect size (95% CI)** | | ***P* of random-effects model** | | **95% PI** | | ***I*^2^(%)** | | **Small-study**  **Effects**  **/ Excess statistical significance** | | **Egger test**  ***P* value** | **Level of evidence** | |
| Grosso, 2017 | **CAFFEINE *** | | | | | | | | | | | | | | | | | | | | | | |
|  | Parkinson disease | | High vs. Low | | 304,980 | | 9 prospective | | RR | | 0.67  (0.57–0.80) | | 0.018 | | N/A | | 46 | | N/A | | N/A | Probable | |
|  | Alzheimer Disease | | High vs. Low | | 6,870 | | 5 observational | | N/SP | | 0.78  (0.50−1.22) | | N/A | | N/A | | 71 | | N/A | | N/A | Limited | |
|  | Dementia | | High vs. Low | | 8,440 | | 5 observational | | N/SP | | 0.72  (0.34−1.51) | | N/A | | N/A | | 76 | | N/A | | N/A | N/A | |
|  | Cognitive Impairment | | High vs. Low | | 5,897 | | 5 observational | | N/SP | | 0.79  (0.61−1.04) | | N/A | | N/A | | 21 | | N/A | | N/A | Limited | |
|  | Cognitive Disorders | | High vs. Low | | 31,479 | | 19 observational | | N/SP | | 0.82  (0.67−1.01) | | N/A | | N/A | | 63 | | N/A | | 0.63 | Possible | |
|  | Cognitive Decline | | High vs. Low | | 10,272 | | 4 observational | | N/SP | | 0.99  (0.70−1.39) | | N/A | | N/A | | 62 | | N/A | | N/A | N/A | |
|  | **COFFEE *** | | | | | | | | | | | | | | | | | | | | | | |
|  | Stroke | | High vs. Low | | 12,030 | | 22 prospective | | RR | | 0.95  (0.84−1.07) | | N/A | | N/A | | 54 | | N/A | | N/A | No associations | |
|  | Parkinson Disease | | N/A | | 187,740 | | 4 prospective | | RR | | 0.70  (0.56−0.88) | | N/A | | N/A | | N/A | | N/A | | N/A | Probable | |
|  | Alzheimer Disease | | High vs. Low | | 15,761 | | 4 prospective | | RR | | 0.73  (0.55−0.97) | | N/A | | N/A | | 0 | | N/A | | N/A | Possible | |
|  | Cognitive Impairment | | High vs. Low | | 6,649 | | 3 prospective | | RR | | 0.78  (0.48−1.26) | | N/A | | N/A | | 49 | | N/A | | N/A | No associations | |
|  | Cognitive Disorders | | High vs. Low | | 29,155 | | 10 prospective | | RR | | 0.97  (0.84−1.10) | | N/A | | N/A | | 25 | | N/A | | N/A | No associations | |
|  | Cognitive Decline | | High vs. Low | | 9,254 | | 4 prospective | | RR | | 1.02  (0.88−1.18) | | N/A | | N/A | | 0 | | N/A | | N/A | No associations | |
|  | Dementia | | High vs. Low | | 12,607 | | 5 prospective | | RR | | 1.08  (0.81−1.44) | | N/A | | N/A | | 28 | | N/A | | N/A | No associations | |
|  | Vascular Dementia | | High vs. Low | | 3,734 | | 1 prospective | | RR | | 1.96  (0.76−5.04) | | N/A | | N/A | | N/A | | N/A | | N/A | N/A | |
|  | Abbreviations: N/SP = not specified (Either OR or RR); N/A = not available; RR = relative risk.  * According to the authors of the paper, *“Whenever more than one meta-analysis was conducted using the same outcome, and the same study design and type of population, concordance was evaluated for the main outcome of interest, including concordance in the direction and magnitude of the association by comparing risk estimatesandconﬁdenceintervals.Forfurtheranalyses,themostrecentormostexhaustivestudy was considered”.*  **# # #** According to the authors of the paper, *“Convincing Level 1a (high): concordance between meta-analyses of RCTs and meta-analyses of observational studies (any); Level 1b (low): meta-analyses of RCTs with results contrary to those from meta-analyses of observational studies (any); Probable Level 2a (high): meta-analyses of prospective studies with no heterogeneity, no potential confounding factors identified, and agreement of results over time and among meta-analyses, including studies with different designs; Level 2b (medium): meta-analyses of prospective studies with no heterogeneity and no potential confounding factors identified; Level 2c (low): meta-analyses of prospective and case-control studies with no heterogeneity and no potential confounding factors identified; Possible Level 3a (high): meta-analyses of prospective studies lacking information on heterogeneity and potential confounding factors; Level 3b (medium): meta-analyses of prospective and case-control studies lacking information on heterogeneity and potential confounding factors; Level 3c (low): meta-analyses of case-control studies or meta-analyses of any other study design with significant heterogeneity (I^2^> 50%) and potential confounding factors; Limited/contrasting Level 4: Limited studies included in meta-analyses (n ≤ 3) or evident contrasting results from meta-analyses with the same level of evidence”.* | | | | | | | | | | | | | | | | | | | | | | |
| Veronese, 2019 | **CHOCOLATE** | | | | | | | | | | | | | | | | | | | | | | |
|  | Stroke | | High  vs. low category | | 322,732 | | 5 observational | | HR | | 0.86  (0.79–0.92) | | 0.001 | | 0.74–0.99 | | 0 | | No / N/A | | 0.008 | IV | |
|  | Abbreviations: N/A = not available; HR = hazard ratio.  **# # #** According to the authors of the paper, *“Evidence class criteria: class I (convincing): statistical significance with P<10−6, more than 1000 cases (or >20 000 participants for continuous outcomes), the largest component study reported statistically significant effect (P<0.05); 95% prediction interval excluded the null; no large heterogeneity (I2 <50%), no evidence of small study effects (P>0.10) and excess significance bias (P>0.10); class II (highly suggestive): statistical significance with P<10−6, more than 1000 cases (or >20 000 participants for continuous outcomes), the largest component study reported statistically significant effect (P<0.05); class III (suggestive): statistical significance with P<10−3, more than 1000 cases (or >20 000 participants for continuous outcomes); class IV (weak): the remaining statistically significant associations with P<0.05. The heterogeneity (I2), Egger’s test, or 95% prediction interval could not be calculated, either because data about the individual component studies were insufficient or because the number of studies included in meta-analyses was less than three. ‡Evidence was reassessed by examining the meta-analyses in depth to verify the eligibility or appropriateness of the data included in analysis and errors were found. When errors and analyses were corrected, the association became non-statistically significant”.* | | | | | | | | | | | | | | | | | | | | | | |
| Poole, 2017 | **COFFEE CONSUMPTION** | | | | | | | | | | | | | | | | | | | | | | |
|  | Cognitive decline | | High vs. low | | 29,155 | | 11 cohort | | RR | | 0.97  (0.85−1.11) | | τ^2^  (0.02) | | N/A | | 24.2% | | N/A | | 0.73 | 6^*^ | |
|  | Stroke | | High vs. low | | 670,223 | | 15 cohort | | RR | | 0.96  (0.83-1.11) | | τ^2^  (0.03) | | N/A | | 53.2 | | N/A | | 0.09 | 8^*^ | |
|  | Glioma | | High vs. low | | 1,669,442 | | 4 cohort | | RR | | 0.98  (0.79-1.23) | | τ^2^  (0.00) | | N/A | | 6.4 | | N/A | | ND | 5^*^ | |
|  | Parkinson disease | | High vs. low consumption | | 894,568 | | 7 cohort | | RR | | 0.64  (0.53–0.76) | | τ^2^  (0.01) | | N/A | | 16 | | N/A | | ND | 5^*^ | |
|  | Parkinson disease | | Consumption of decaffeinated coffee | | 251,300 | | 4  observational | | RR | | 0.94 ⸹  (0.78−1.12) | | N/A | | N/A | | N/A | | N/A | | N/A | 6^*^ | |
|  | Parkinson disease | | Any vs. No coffee consumption | | 719,187 | | 6 cohort | | RR | | 0.64  (0.53−0.77) | | τ^2^  (0.02) | | N/A | | 29 | | N/A | | ND | 7^*^ | |
|  | Parkinson disease | | Consumption of one extra cup of coffee  a day | | 187,281 | | 4 cohort | | RR | | 0.88  (0.77−1.00) | | N/A | | N/A | | N/A | | N/A | | N/A | 4^*^ | |
|  | Alzheimer disease | | Consumption of one extra cup of coffee  a day | | N/A | | 2 cohort | | RR | | 1.02  (0.95−1.08) | | N/A | | N/A | | 16 | | N/A | | N/A | 6^*^ | |
|  | Alzheimer disease | | Any vs. No coffee consumption | | 5,497 | | 2 cohort | | RR | | 0.73  (0.54–0.99) | | τ^2^  (0.00) | | N/A | | 0 | | N/A | | ND | 3^*^ | |
|  | Abbreviations: NP = not published; ND = not done; N/A = not available; ⸹ Effect model not published.  ^*^**# # #** According to the authors of the paper, *AMSTAR: A Measurement tool to Assess Systematic Reviews; # Q1: A-priori design; Q2: Duplicate study selection and data extraction; Q3: Search comprehensiveness; Q4: Inclusion of gray literature; Q5: Included and excluded studies provided; Q6: Characteristics of the included studies provided; Q7: Scientific quality of the primary studies assessed and documented; Q8: Scientific quality of included studies used appropriately in formulating conclusions; Q9: Appropriateness of methods used to combine studies’ findings; Q10: Likelihood of publication bias was assessed; Q11: Conflict of interest-potential sources of support were clearly acknowledged in both the systematic review and the included studies”.* | | | | | | | | | | | | | | | | | | | | | | |
| Veronese, 2018 | **DIETARY FIBER** | | | | | | | | | | | | | | | | | | | | | | |
|  | Stroke | | Highest vs. Lowest category | | 325,707 | | 14 prospective | | RR | | 0.828  (0.740–0.926) | | 9 × 10^–4^ | | 0.61–1.12 | | 38.7 | | Yes/0.59 | | N/A | III | |
|  | Abbreviations: N/A = not available; RR = relative risk.  **# # #** According to the authors of the paper, *“Evidence class criteria: class I (convincing): statistical significance with P<10−6, more than 1000 cases (or >20 000 participants for continuous outcomes), the largest component study reported statistically significant effect (P<0.05); 95% prediction interval excluded the null; no large heterogeneity (I2 <50%), no evidence of small study effects (P>0.10) and excess significance bias (P>0.10); class II (highly suggestive): statistical significance with P<10−6, more than 1000 cases (or >20 000 participants for continuous outcomes), the largest component study reported statistically significant effect (P<0.05); class III (suggestive): statistical significance with P<10−3, more than 1000 cases (or >20 000 participants for continuous outcomes); class IV (weak): the remaining statistically significant associations with P<0.05. The heterogeneity (I2), Egger’s test, or 95% prediction interval could not be calculated, either because data about the individual component studies were insufficient or because the number of studies included in meta-analyses was less than three. ‡Evidence was reassessed by examining the meta-analyses in depth to verify the eligibility or appropriateness of the data included in analysis and errors were found. When errors and analyses were corrected, the association became non-statistically significant”.* | | | | | | | | | | | | | | | | | | | | | | |
| McRae, 2016 | **DIETARY WHOLE GRAINS** | | | | | | | | | | | | | | | | | | | | | | |
|  | Stroke  (Fang et al, 2015) | | High vs. Low | | 247,487 | | 6 prospective | | RR | | 0.86  (0.73-0.99) | | <0.05 | | N/A | | 0 | | N/A | | 0.84 | IV | |
|  | Stroke  (Chen et al, 2016) | | High vs. Low | | 204,895 | | 5 cohort | | RR | | 0.92  (0.72-1.17) | | NS | | N/A | | 53 | | N/A | | 0.96 | NS | |
|  | Abbreviations: NS = non-significant; N/A = not available; RR = relative risk.  **# # #** Due to lack of pragmatic approach, level of evidence was computed as: *“Evidence class criteria: class I (convincing): statistical significance with P<10−6, more than 1000 cases (or >20 000 participants for continuous outcomes), the largest component study reported statistically significant effect (P<0.05); 95% prediction interval excluded the null; no large heterogeneity (I2 <50%), no evidence of small study effects (P>0.10) and excess significance bias (P>0.10); class II (highly suggestive): statistical significance with P<10−6, more than 1000 cases (or >20 000 participants for continuous outcomes), the largest component study reported statistically significant effect (P<0.05); class III (suggestive): statistical significance with P<10−3, more than 1000 cases (or >20 000 participants for continuous outcomes); class IV (weak): the remaining statistically significant associations with P<0.05. The heterogeneity (I^2^), Egger’s test, or 95% prediction interval could not be calculated, either because data about the individual component studies were insufficient or because the number of studies included in meta-analyses was less than three. ‡Evidence was reassessed by examining the meta-analyses in depth to verify the eligibility or appropriateness of the data included in analysis and errors were found. When errors and analyses were corrected, the association became non-statistically significant”.* | | | | | | | | | | | | | | | | | | | | | | |
| Galbete, 2018 | **MEDITERRANEAN DIET** | | | | | | | | | | | | | | | | | | | | | | |
|  | Unspecified stroke | | High vs. low | | 107,074 | | 5 cohort | | RR | | 0.77  (0.67−0.90) | | N/A | | 0.61–0.98 | | 0 | | N/A | | N/A | N/A | |
|  | Ischemic stroke | | High vs. low | | 181,295 | | 5 cohort | | RR | | 0.82  (0.73−0.92) | | N/A | | 0.67−1.00 | | 0 | | N/A | | N/A | N/A | |
|  | Hemorrhagic stroke | | High vs. low | | 178,727 | | 4 cohort | | RR | | 1.01  (0.74−1.37) | | N/A | | 0.36−2.82 | | 36 | | N/A | | N/A | N/A | |
|  | Mild cognitive impairment incidence**^@^** | | High vs. low | | 27,567 | | 5 cohort | | RR | | 0.83  (0.75–0.93) | | 0.001 | | 0.7–1.0 | | N/A | | N/A | | N/A | IV | |
|  | Alzheimer disease | | High vs. low | | 7,609 | | 5 cohort | | RR | | 0.60  (0.48–0.77) | | <.0001 | | 0.41–0.89 | | 0 | | N/A | | N/A | IV | |
|  | Dementia | | High vs. low | | 9,811 | | 3 cohort | | RR | | 1.07  (0.81−1.42) | | 0.063 | | 0.17−6.61 | | 0 | | N/A | | N/A | NS | |
|  | Mild cognitive impairment incidence/Dementia | | High vs. low | | 6,294 | | 5 cohort | | RR | | 0.69  (0.57−0.84) | | <0.001 | | 0.49−0.96 | | 1 | | N/A | | NS | IV | |
|  | Mild cognitive impairment incidence | | High vs. low | | 2,674 | | 2 cohort | | RR | | 0.73  (0.56−0.96) | | 0.02 | | N/A | | 0 | | N/A | | N/A | IV | |
|  | Alzheimer disease incidence | | High vs. low | | 3,439 | | 2 cohort | | HR | | 0.64  (0.46−0.89) | | 0.007 | | N/A | | 0 | | N/A | | N/A | IV | |
|  | Stroke **^@^** | | High vs low | | 152,843 | | 5 cohort | | RR | | 0.84  (0.74−0.95) | | 0.007 | | 0.68−1.03 | | 0 | | N/A | | N/A | IV | |
|  | Abbreviations: NS = non-significant; N/A = not available; RR = relative risk  **# # # Due to lack of pragmatic approach, level of evidence was computed as:** *“Evidence class criteria: class I (convincing): statistical significance with P<10−6, more than 1000 cases (or >20 000 participants for continuous outcomes), the largest component study reported statistically significant effect (P<0.05); 95% prediction interval excluded the null; no large heterogeneity (I2 <50%), no evidence of small study effects (P>0.10) and excess significance bias (P>0.10); class II (highly suggestive): statistical significance with P<10−6, more than 1000 cases (or >20 000 participants for continuous outcomes), the largest component study reported statistically significant effect (P<0.05); class III (suggestive): statistical significance with P<10−3, more than 1000 cases (or >20 000 participants for continuous outcomes); class IV (weak): the remaining statistically significant associations with P<0.05. The heterogeneity (I^2^), Egger’s test, or 95% prediction interval could not be calculated, either because data about the individual component studies were insufficient or because the number of studies included in meta-analyses was less than three. ‡Evidence was reassessed by examining the meta-analyses in depth to verify the eligibility or appropriateness of the data included in analysis and errors were found. When errors and analyses were corrected, the association became non-statistically significant”.* | | | | | | | | | | | | | | | | | | | | | | |
| Dinu, 2017 | **MEDITERRANEAN DIET** | | | | | | | | | | | | | | | | | | | | | | |
|  | **1. OBSERVATIONAL STUDIES** | | | | | | | | | | | | | | | | | | | | | | |
|  | Stroke**^@^** | | High vs low | | 152,843 | | 5 cohort | | RR | | 0.84  (0.74−0.95) | | 0.007 | | 0.68−1.03 | | 0 | | N/A | | N/A | Weak | |
|  | Stroke | | High vs. low | | 296 | | 2 case -control | | RR | | 0.20  (0.10−0.41) | | <0.0001 | | 0.09−0.46 | | 0 | | N/A | | N/A | Weak | |
|  | Stroke | | High vs. low | | 10,847 | | 5 cross-sectional | | RR | | 0.83  (0.66−1.06) | | 0.13 | | 0.51−1.37 | | 13 | | N/A | | N/A | No evidence | |
|  | Stroke | | High vs. low | | 159,995 | | 5 cohort | | RR | | 0.76  (0.60−0.96) | | 0.02 | | 0.36−1.59 | | 52 | | N/A | | N/A | Weak | |
|  | Neurodegenerative diseases | | 2-point increase | | 136,235 | | 5 cohort | | RR | | 0.87  (0.81−0.94) | | 0.0005 | | 0.77−0.98 | | 0 | | N/A | | N/A | Convincing | |
|  | Neurodegenerative diseases | | High vs. low | | 34,168 | | 9 cohort | | RR | | 0.79  (0.70−0.90) | | 0.0003 | | 0.60−1.04 | | 22 | | N/A | | N/A | Highly suggestive | |
|  | Cognitive impairment | | High vs. low | | 5,916 | | 4 cohort | | RR | | 0.72  (0.58−0.88) | | 0.001 | | 0.45−1.13 | | 0 | | N/A | | N/A | Suggestive | |
|  | Cognitive impairment | | High vs. low | | 6,652 | | 5 cohort | | HR | | 0.67  (0.55−0.81) | | <0.0001 | | 0.45−0.91 | | 0 | | N/A | | N/A | Suggestive | |
|  | Cognitive impairment | | 1-point increase | | 6,878 | | 5 cohort | | HR | | 0.92  (0.88−0.97) | | 0.0008 | | 0.85−1.00 | | 0 | | N/A | | N/A | Suggestive | |
|  | Cognitive impairment | | High vs. low | | 1,880 | | 1 case-control | | RR | | 0.31  (0.16−0.59) | | 0.004 | | NE^◊^ | | NE^◊^ | | N/A | | N/A | Weak | |
|  | Cognitive impairment | | High vs. low | | 3,345 | | 3 cohort | | RR | | 0.52  (0.22−1.22) | | 0.13 | | 0.01−22.20 | | 88 | | N/A | | N/A | No evidence | |
|  | Cognitive impairment**^@^** | | High vs. low | | 27,567 | | 5 cohort | | RR | | 0.83  0.75−0.93) | | 0.001 | | 0.66−1.05 | | 0 | | N/A | | N/A | Highly suggestive | |
|  | Alzheimer disease | | High vs. low | | 6,111 | | 5 cohort | | RR | | 0.60  (0.48–0.77) | | <0.0001 | | 0.44–0.82 | | 0 | | N/A | | N/A | Convincing | |
|  | Dementia | | High vs. low | | 8,174 | | 5 cohort | | RR | | 0.69  (0.57–0.84) | | 2 x 10^−5^ | | 0.51–0.95 | | 1 | | N/A | | N/A | Convincing | |
|  | Dementia | | High vs. low | | 8,873 | | 3 cohort | | RR | | 1.07  (0.81−1.42) | | 0.63 | | 0.58−1.97 | | 0 | | N/A | | N/A | No evidence | |
|  | 1. **RANDOMIZED CONTROL TRIALS** | | | | | | | | | | | | | | | | | | | | | | |
|  | Stroke | | High vs. low | | 10,688 | | 2 randomized control trials | | RR | | 0.64  (0.47−0.86) | | 0.004 | | NE^◊^ | | 0% | | N/A | | N/A | Weak | |
|  | Stroke | | N/A | | 8,052 | | 2 randomized control trials | | RR | | 0.64  (0.34−1.20) | | 0.16 | | NE^◊^ | | 6% | | N/A | | N/A | No evidence | |
|  | Abbreviations: N/A = not available; RR = relative risk.  ^◊^ According to the authors, *“NE = not estimable because less than 3 studies were available for each meta-analysis”.*  **# # #** According to the authors of the paper, *“Convincing evidence class: significance threshold reached at P≤0.001 for both random- and fixed effects calculation;>1000 cases (or >5,000 total participants if the metric was continuous); not large heterogeneity between studies(I^2^<50%); 95% PI excluding the null value; no evidence of small study effects (if it could be tested); Highly suggestive evidence: significance threshold reached at P≤0.001 for both random- and fixed effects calculation; >1000cases (or >5000 total participants if the metric was continuous);not considerable heterogeneity between studies (I^2^=50–75%);Suggestive evidence: significance threshold reached at P≤0.001for random-effect calculation; 500–1000 cases (or 2500–5000total participants if the metric was continuous); Weak evidence: significance threshold reached at P≤ 0.05 for random effects calculation; No evidence: significance threshold not reached (P>0.05).* | | | | | | | | | | | | | | | | | | | | | | |
| Posadzki, 2018 | **MELATONIN** | | | | | | | | | | | | | | | | | | | | | | |
|  | Dementia (cognition as health outcome) ^≠^ | | N/A | | 121 | | 2 randomized control trials | | MD | | –2.64  (–5.98 to 0.71) | | 0.123 | | N/A | | 68.6 | | − / No | | N/A | NS | |
|  | Dementia  (mood and behavior as health outcome) | | N/A | | 150 | | 3 randomized control trials | | MD | | 0.18  (−0.73 to 1.10) | | 0.698 | | −5.76 to −6.12 | | 0 | | No / No | | N/A | NS | |
|  | Abbreviations: NS = non-significant; N/A = not available; RR = relative risk; MD = mean difference.  **≠** According to authors, *“The 95% prediction interval and the evidence of small-study effects were calculated for those MAs where ≥3 studies combined (it cannot be calculated for less than three studies as degrees of freedom will be zero for two studies and negative for one study)”*.  **# # #** *Due to lack of pragmatic approach, level of evidence was computed as NS because p<.005.* | | | | | | | | | | | | | | | | | | | | | | |
|  | **SERUM URIC ACID** | | | | | | | | | | | | | | | | | | | | | | |
| Li, 2017 | 1. **OBSERVATIONAL STUDIES** | | | | | | | | | | | | | | | | | | | | | | |
|  | Diabetic peripheral neuropathy | | Hyper vs. normal | | 4,097 | | 5 cohort or case-control | | RR | | 2.83  (2.13−3.76) | | 2.91 x 10^−12^ | | 1.05−7.62 | | 78 | | 0.93 | | 0.94 | IV | |
|  | Stroke mortality | | Highest vs. lowest SUA category | | 1,017,810 | | 9 prospective cohort | | aRR | | 1.32  (1.23−1.41) | | 1.11E−14 | | 1.13−1.56 | | 30 | | NP | | 0.92 | I^∩^ | |
|  | Alzheimer disease | | General SUA (mg/dl) | | 3,617 | | 21  cohort or case-control | | MD to OR | | 0.29  (0.11−0.76) | | 0.012 | | 0.01−8.97 | | 97 | | NP | | 0.30 | IV | |
|  | Dementia or cognitive impairment | | General SUA (mg/dl) | | 7,021 | | 31  cohort or case-control | | SMD to OR | | 0.58  (0.41−0.83) | | 0.003 | | 0.08−4.48 | | 89 | | 0.004 | | 0.01 | IV | |
|  | Parkinson incidence | | Hyper vs.  normal | | 33,185 | | 6  cohort and nested case-control | | RR | | 0.65  (0.43–0.97) | | 0.04 | | 0.24–1.77 | | 42 | | NP | | 0.39 | IV | |
|  | Multiple sclerosis | | General SUA  (mg/dl) | | 2,216 | | 10 case-control | | SMD to OR | | 0.49  (0.27–0.87) | | 0.02 | | 0.05–4.96 | | 92 | | NP | | 0.11 | IV | |
|  | Neuromyelitis Optica | | General SUA  (mg/dl) | | 1,137 | | 3 case-control | | SMD to OR | | 0.22  (0.10–0.45) | | 9.07 x10^−5^ | | 0.02–3.14 | | 82 | | 0.93 | | 0.65 | IV | |
|  | Amyotrophic lateral sclerosis (ALS) | | Patients with ALS vs. Controls SUA (mg/dl) | | 826 | | 3 case-control | | Hedge’s G to OR | | 0.21  (0.14–0.32) | | 6.3 × 10^–13^ | | 0.04–1.05 | | 51 | | NP | | 0.43 | IV | |
|  | Abbreviations: NP = not pertinent (because the number of expected significant studies was larger than the number of observed significant studies); RR = relative risk; OR = odds ratio; aRR = adjusted relative risk; MD = mean difference; SMD = standardized mean difference; SUA = serum uric acid.  **Other environmental factors and biomarkers with non-significant associations in “OBSERVATIONAL STUDIES”: Stroke; Vascular Dementia; Mild Cognitive Impairment**  **^∩^**According to the authors, *“Evidence was re-assessed by examining the meta-analyses in depth to verify the eligibility of appropriateness of the data included in analysis, and errors were found. When errors and analyses were corrected, the association became non-statistically significant”*.  **# # #** According to the authors of the paper, *“Evidence class criteria: class I (convincing): statistical significance with P<10−6, more than 1000 cases (or >20 000 participants for continuous outcomes), the largest component study reported statistically significant effect (P<0.05); 95% prediction interval excluded the null; no large heterogeneity (I^2^<50%), no evidence of small study effects (P>0.10) and excess significance bias (P>0.10); class II (highly suggestive): statistical significance with P<10−6, more than 1000 cases (or >20 000 participants for continuous outcomes), the largest component study reported statistically significant effect (P<0.05); class III (suggestive): statistical significance with P<10−3, more than 1000 cases (or >20 000 participants for continuous outcomes); class IV (weak): the remaining statistically significant associations with P<0.05. The heterogeneity (I^2^), Egger’s test, or 95% prediction interval could not be calculated, either because data about the individual component studies were insufficient or because the number of studies included in meta-analyses was less than three. ‡Evidence was reassessed by examining the meta-analyses in depth to verify the eligibility or appropriateness of the data included in analysis and errors were found. When errors and analyses were corrected, the association became non-statistically significant”.* | | | | | | | | | | | | | | | | | | | | | | |
|  | 1. **RANDOMISED CONTROLLED TRIALS** | | | | | | | | | | | | | | | | | | | | | | |
|  | Death or severe neurodevelopmental disabilities  (All infants) | | Allopurinol as SUA lowering treatment | | 110 | | 3 randomized controlled trials | | RR | | 0.85  (0.63−1.15) | | 0.29 | | 0.12−5.98 | | 0 | | NP | | 0.12 | NS | |
|  | Death or severe developmental disabilities  (Infants with severe hypoxic-ischemic encephalopathy) ∞ | | Allopurinol as SUA lowering treatment | | 41 | | 2 randomized controlled trials | | RR | | 0.93  (0.67−1.30) | | 0.68 | | NA | | NA | | NP | | NA | NS | |
|  | Severe quadriplegia | | Allopurinol as SUA lowering treatment | | 73 | | 3 randomized controlled trials | | RR | | 0.58  (0.27−1.26) | | 0.17 | | 0.01−86.99 | | 0 | | NP | | 0.69 | NS | |
|  | Seizures in neonatal period | | Allopurinol | | 114 | | 3 randomized controlled trials | | RR | | 0.98  (0.84−1.15) | | 0.81 | | 0.35−2.79 | | 0 | | NP | | 0.15 | NS | |
|  | NA = not applicable (did not calculate with only 2 studies); NP = not pertinent (because the number of expected significant studies was larger than the number of observed significant studies); RR = relative risk; NS = non-significant.  **Other environmental factors and biomarkers with non-significant association in “RANDOMISED CONTROLLED TRIALS”**  None related to neurological/ brain-related disorders  SUA: Serum Uric Acid; NA=not applicable (did not calculate with only 2 studies); NP=not pertinent (because the number of expected significant studies was larger than the number of observed significant studies).  **∞** According to the authors, *“the strength of evidence was graded based on the evidence-based practice center approach (conceptually similar to GRADE ranking system): recurrence of nephrolithiasis (with allopurinol, thiazides, or citrates treatment) was all considered with moderate evidence in original meta-analyses”*.  **# # #** Due to lack of pragmatic approach, *level of evidence was computed as NS because p<.005.* | | | | | | | | | | | | | | | | | | | | | | |
|  | 1. **MENDELIAN RANDOMISATION STUDIES** | | | | | | | | | | | | | | | | | | | | | | |
|  | **Neurological Disorder** | | **No/No of Events (No of Studies) ^∟^** | | **Genetic Instruments (GI)** | | **SUA variance (R^2^) explained by GI (%)** | | **Type of metric** | | **Estimate of effect (95% CI)** | | ***P* value** | | **Statistical power**^£^ | | | | | | | | |
|  | Ischemic Stroke | | 82,091 / 14,779 (2) ^≠^ | | Genetic risk score of 14 SUA related SNPs | | 3.1 | | OR | | 0.99  (0.82−1.12) | | 0.93 | | 0.05 | | | | | | | | |
|  | Parkinson Disease | | 1,815 / 1,061 | | Genetic risk score of 8 SUA related SNPs | | NA | | OR | | 1.55  (1.10−2.18) | | 0.01 | | 0.59^⁋^ | | | | | | | | |
|  | Age of onset of Parkinson Disease | | 666 (3) ^≠^ | | 4 SNPs in *SCL2A9* | | NA | | β | | Null after multiple testing correction | | | |  | | | | | | | | |
|  |  |  |  |  | rs737267 | | NA | |  |  | 3.10  (0.17 to 6.03) | | 0.04 | | NA | | | | | | | | |
|  |  |  |  |  | rs6449213 | | NA | |  |  | −1.18  (−4.96 to 2.59) | | 0.54 | |  |  |  |  |  |  |  |  |  |
|  |  |  |  |  | rs1014290 | | NA | |  |  | −4.56  (−8.13 to −1.00) | | 0.01 | |  |  |  |  |  |  |  |  |  |
|  |  |  |  |  | rs733175 | | NA | |  |  | 3.59 (0.67 to 6.51) | | 0.02 | |  |  |  |  |  |  |  |  |  |
|  | Memory performance | | 1,091 | | 4 SNPs in *SCL2A9*  (“European Population 1”) | | NA | | β | | Overall *p*<0.05 | | | | NA | | | | | | | | |
|  |  |  | 1,066 | | 4 SNPs in *SCL2A9*  (“European Population 2”) | | NA | | β | | Overall *p*> 0.05 | | | |  |  |  |  |  |  |  |  |  |
|  | Abbreviations: NA = not available; OR= odds ratio; RR = relative risk; NS = non-significant; β = regression coefficient; SNPs, single nucleotide polymorphisms; SUA, serum uric acid  **^∟^**According to the authors, *“If the outcomes were reported from Mendelian randomization analysis with pooling multiple studies, the number of studies included in pooled analysis was displayed in brackets”.*  ^£^According to the authors, *“When Mendelian randomisation studies did not provide other necessary information for calculation (eg, standard deviation of serum uric acid levels, standard deviation of outcomes, or the number of cases), the statistical power was not calculated (reported as NA)”.*  ^≠^According to the authors, *“If the outcomes were reported from Mendelian randomisation analysis with pooling multiple studies, the number of studies included in pooled analysis was displayed in brackets”.*  ^⁋^According to the authors, *“The statistical power was a crude estimation, as the Mendelian randomisation studies failed to report R2; we used the extrapolated R2 from other Mendelian randomisation studies that used the same genetic variants as instruments for calculation”.* | | | | | | | | | | | | | | | | | | | | | | |
| Theodoratou, 2014 | **VITAMIN D (25−OHD)** | | | | | | | | | | | | | | | | | | | | | | |
|  |  |  | |  | |  | |  | |  | |  | |  | |  | |  | |  | | |  |
|  | Alzheimer disease | High vs low | | 1,005 | | 7  observational | | SMD  (to OR) | | 0.08  (0.01-0.63) | | 0.02ƪ | | N/A | | 98  (97−98) | | 3.8x10^−6^ | | 0.32 | | | No conclusion |
|  | Cognition | High vs low | | 9,004 | | 7  observational | | OR | | 0.42  (0.34-0.53) | | 2.2 ×  10^–13^ƪ | | N/A | | 56  (0−79) | | 0.47 | | 0.16 | | | Suggestive |
|  | Stroke | High vs low | | 39,095 | | 7 observational | | RR | | 0.61  (0.50-0.75) | | 1.8 ×  10^–6^  ƪ | | N/A | | 0  (0−58) | | N/A | | 0.94 | | | Suggestive |
|  | Ischemic Stroke | High vs low | | 26,596 | | 4 observational | | HR | | 0.66  (0.55-0.80) | | 2.1 x 10^-5^  ƪ | | N/A | | 71  (0−86) | | 0.76 | | 0.63 | | | Suggestive |
|  | Ischemic Stroke | High vs low | | 31,858 | | 5 observational | | OR | | 0.52  (0.44-0.61) | | 2.3 x 10^-14^  ƪ | | N/A | | 0  (0−64) | | 0.81 | | 0.97 | | | Suggestive |
|  | Abbreviations: N/A = not available; RR = relative risk; OR = odds ratio; HR = hazard ratio; SMD = standardized mean difference.  ƪ (based on Altman and Bland 2011)  **# # #** According to the authors, *“Convincing: Evidence existed from both observational studies and randomised controlled trials (RCTs), and association/effect was of the same direction, statistically significant at P≤0.001, and free from bias; Probable: Evidence existed from both observational studies and RCTs, and association/effect was of the same direction and statistically significant at P≤0.001, but excess significance could not be tested; or evidence existed from RCTs and effect was statistically significant at P≤0.001 and with no contrary results from observational data (that is, systematic reviews, if any exist, are also definitive or suggestive and meta-analyses of observational studies, if any exist, are in the same direction); Suggestive: Evidence from RCTs with an effect at 0.001≤ P≤0.05 and with no contrary results from observational data (same as above); or evidence from meta-analyses of observational studies showing an association at P≤0.001, with no contrary results from randomized data (that is, meta-analysis of RCTs, if present, have effects in the same direction) and, if it could be tested, no evidence of small study effects (P≥0.10), not very large heterogeneity (I^2^≤75%), no evidence for excess significance, based on cumulative evidence of more than 500 disease events (or more than 5000 total participants if type of metric was continuous); No conclusion: Not enough evidence from observational studies or RCTs to draw conclusion; Substantial effect unlikely: Evidence from observational studies or RCTs enough to conclude that a substantial effect is unlikely based on the magnitude and the significance level”.* | | | | | | | | | | | | | | | | | | | | | | |
| @ : Studies overlapping | | | | | | | | | | | | | | | | | | | | | | | |

| **Table S1.b.: Specific neurological diseases versus different protective and risk factors** | | | | | | | | | | | | |
| --- | --- | --- | --- | --- | --- | --- | --- | --- | --- | --- | --- | --- |
| **Reference** | **Outcome** | **Level of comparison** | **Total number of participants in each meta-analysis** | **Number of primary studies** | **Effect size metric** | **Random-effects summary effect size (95% CI)** | ***P* value** | **95% PI** | ***I*^2^(%)** | **Small- study**  **Effects**  **/ Excess statistical significance** | **Egger test *P* value** | **Level of evidence** |
| Belbasis, 2016 | **AMYOTROPHIC LATERAL SCLEROSIS** | | | | | | | | | | | |
|  | Serum uric acid | High vs.  Low values | 826 | 3 observational | OR^#^ | 0.31  (0.18–0.52) | 1.27 × 10^–5^ | 0.001–1.37 | 71.9 | 0.45 | 0.117 | Weak |
|  | Serum HDL | High vs. Low values | 5,565 | 8 observational | OR^#^ | 1.05  (0.75−1.46) | 7.94 x 10^−1^ | 0.33−3.27 | 87.0 | 8.00 × 10^–5^ | 0.548 | NS |
|  | Serum LDL | High vs. Low values | 5,565 | 8 observational | OR^#^ | 0.95  (0.44−2.05) | 9.02 x 10^−1^ | 0.06−15.85 | 97.6 | 3.18 × 10^–3^ | 0.862 | NS |
|  | Serum total cholesterol | High vs. Low values | 5,565 | 8 observational | OR^#^ | 0.99  (0.59−1.68) | 9.96 x 10^−1^ | 0.15−6.49 | 94.8 | 4.10 × 10^–4^ | 0.772 | NS |
|  | Serum triglycerides | High vs. Low values | 3,162 | 6 observational | OR^#^ | 0.98  (0.82−1.17) | 8.37 x 10^−1^ | 0.64−1.51 | 35.1 | NP^&^ | 0.844 | NS |
|  | β-Carotene intake | 2,500 μg/day increase | 1,053,575 | 9 observational | RR | 0.92  (0.87–0.97) | 2.24 × 10^–3^ | 0.86–0.98 | 0 | 0.59 | 0.917 | Suggestive |
|  | n-3 fatty acids intake | 1 g/day increase | 1,056,837 | 8 observational | RR | 0.71  (0.59–0.85) | 1.95 × 10^–4^ | 0.49–1.01 | 0 | NP^&^ | 0.056^ | Weak |
|  | Head injury | Exposed vs. not exposed | 6,470 | 8 observational | OR | 1.65  (1.09−2.51) | 1.80 x 10^−2^ | 0.59−4.64 | 42.6 | NP^&^ | 0.751 | Suggestive |
|  | Statins | Exposed vs. not exposed | 129,170 | 3 observational | RR | 1.15  (0.78−1.70) | 4.85 x 10^−1^ | 0.01−1.06 | 74.2 | 0.03 | 0.767 | NS |
|  | Smoking | Ever smokers vs. never smokers | 3,020,299 | 20 observational | RR | 1.12  (0.98−1.27) | 1.03 x 10^−1^ | 0.70−1.77 | 57.6 | NP^&^ | 0.734 | NS |
|  | Farming | Exposed vs. not exposed | 2,614,659 | 10 observational | OR | 1.42  (1.17–1.73) | 3.75 × 10^–4^ | 0.90–2.26 | 42.1 | 0.24 | 0.857 | Suggestive |
|  | Pesticides | Exposed vs. not exposed | 3,725,678 | 15 observational | OR | 1.44  (1.22–1.70) | 2.04 × 10^–5^ | 0.94–2.20 | 41.2 | 0.08 | 0.163 | Weak |
|  | Rural living | Exposed vs. not exposed | 1,591 | 5 observational | OR | 1.25  (0.84−1.88) | 2.76 x 10^−1^ | 0.35−4.54 | 59.7 | 0.19 | 0.618 | NS |
|  | Other heavy metals | Exposed vs. not exposed | 1,015 | 4 observational | OR | 2.13  (1.33–3.41) | 1.60 × 10^–3^ | 0.48–9.52 | 26.0 | NP^&^ | 0.368 | Suggestive |
|  | Lead | Exposed vs. not exposed | 2,772 | 9 observational | OR | 1.81  (1.39–2.35) | 8.72 × 10^–6^ | 1.14–2.88 | 12.7 | NP^&^ | 0.110 | Convincing |
|  | Extremely low frequency  electromagnetic fields | Exposed vs. not exposed | 9,915,540 | 17 observational | OR | 1.29  (1.03–1.62) | 0.03 | 0.64–2.59 | 58.9 | NP^&^ | 0.034 | Weak |
|  | Abbreviations NP = not pertinent (because the number of expected significant studies was larger than the number of observed significant studies); OR = odds ratio; NS = non-significant; HDL = high density lipoprotein; LDL = low density lipoprotein  OR^#^: According to the authors of the paper, *“Weighted mean difference transformed to OR”*.  ^: According to the authors of the umbrella review, *“In the annotated paper, the Egger test was statistically significant (p < 0.10) and the largest study has more conservative effect size compared to the summary effect size under random effects, denoting the existence of small-study effects”*.  **# # #** According to the author of the paper, *“we characterized as convincing the associations that met the following criteria: had significance according to random-effects meta-analysis less than 0.001; were based on greater than 1,000 cases; had between-study heterogeneity that was not large (I ^2^ <50%)and a 95% PI excluded the null value; and had not evidence of small-study effects and excess significance bias. We characterized as suggestive the associations that presented not large between study heterogeneity and had not evidence of small-study effects and excess significance bias. The remaining significant associations were characterized as weak”.* | | | | | | | | | | | |
| Bellou, 2017 | **ALL TYPES OF DEMENTIA** | | | | | | | | | | | |
|  | Alcohol drinking | Light or moderate vs. never | 17,405 | 7 observational | RR | 0.74  (0.61–0.91) | 3.9 × 10^–3^ | 0.43–1.28 | 52.6 | No/Yes | N/A | Weak |
|  | Physical activity | High level vs. Low level | 39,819 | 21 observational | RR | 0.76  (0.66–0.86) | 1.9 × 10^–5^ | 0.49–1.15 | 68.9 | Yes/Yes | N/A | Suggestive |
|  | Depression  at any age | Diseased vs. not diseased | 441,491 | 33 observational | RR | 1.99  (1.84–2.16) | 8.0 × 10^–62^ | 1.65–2.40 | 27.8 | No/No | N/A | Convincing |
|  | Early-life depression | Diseased vs. not diseased | 28,383 | 9 observational | RR | 1.63  (1.27–2.11) | 1.5 × 10^–4^ | 1.01–2.64 | 16.2 | No/No | N/A | Suggestive |
|  | Late-life depression | Diseased vs. not diseased | 51,353 | 25 observational | RR | 1.85  (1.67–2.05) | 3.1 × 10^–32^ | 1.66–2.06 | 0 | No/No | N/A | Convincing |
|  | Type 2 diabetes mellitus | Diseased vs. not diseased | 1,141,157 | 22 observational | RR | 1.60  (1.43–1.79) | 5.4× 10^–17^ | 1.05–2.44 | 72.3 | No/No | N/A | Highly suggestive |
|  | Frequency of social contacts | Low level vs.  high level | 15,762 | 8 observational | RR | 1.57  (1.32−1.85) | 1.9 x 10^−7^ | 1.27−1.93 | 0 | No/No | N/A | Convincing |
|  | Loneliness | High vs. Low | 3,252 | 3 observational | RR | 1.58  (1.19–2.09) | 1.5 × 10^–3^ | 0.25–9.78 | 0 | No/No | N/A | Weak |
|  | Social participation | Low level vs.  high level | 7,714 | 6 observational | RR | 1.41  (1.13–1.75) | 2.0 × 10^–3^ | 0.85–2.34 | 31.2 | Yes/Yes | N/A | Weak |
|  | Antihypertensive drugs | Ever vs. never | 1,523,417 | 11 observational | HR | 0.84  (0.75–0.94) | 1.7 × 10^–3^ | 0.60–1.16 | 73.4 | Yes/No | N/A | Weak |
|  | Midlife BMI | Obese  vs. normal weight | 30,319 | 5 observational | RR | 1.91  (1.40–2.62) | 5.1× 10^–5^ | 0.74–4.93 | 53.5 | No/No | N/A | Suggestive |
|  | Education | Low level vs.  High level | 87,243 | 23 observational | RR | 1.88  (1.51−2.33) | 1.2 x 10^−8^ | 0.69−5.14 | 89.6 | No/Yes | N/A | Suggestive |
|  | Late-life BMI | Obese vs. normal weight | 8,453 | 4 observational | RR | 0.83  (0.74−0.94) | 3.0 x 10^−3^ | 0.64−1.09 | 0 | No/No | N/A | Weak |
|  | Mild traumatic  brain injury | Exposed vs. not exposed | 30,362 | 23 observational | OR | 1.35  (1.01−1.78) | 0.040 | 0.38−4.75 | 85 | No/No | N/A | Weak |
|  | Statins | Ever  vs. never users | 4,632,816 | 12 observational | RR | 0.83  (0.76−0.91) | 1.3 x 10^−4^ | 0.66−1.04 | 63.2 | Yes/No | N/A | Suggestive |
|  | Atrial fibrillation | Diseases vs. not diseased | 73,887 | 9 observational | HR | 1.36  (1.12−1.65) | 2.0 x 10^−3^ | 0.82−2.26 | 52.5 | No/No | N/A | Weak |
|  | Tooth loss | High vs. low | 19,817 | 10 observational | RR | 1.56  (1.25−1.96) | 1.1 x 10^−4^ | 0.89−2.75 | 44.8 | No/Yes | N/A | Weak |
|  | Rheumatoid arthritis | Diseases vs. not diseased | 3,063,289 | 4 observational | RR | 1.58  (1.04−2.40) | 0.031 | 0.25−10.14 | 93.4 | No/No | N/A | Weak |
|  | Benzodiazepines use | Ever vs. never smokers | 41,722 | 5 observational | RR | 1.49  (1.30−1.72) | 2.7 x 10^−8^ | 1.03−2.17 | 35.1 | No/No | N/A | Convincing |
|  | Smoking | Ever vs. never smokers | 945,014 | 27 observational | RR | 1.13  (1.05−1.22) | 1.2 x 10^−3^ | 0.87−1.47 | 47.1 | No/Yes | N/A | Weak |
|  | **VASCULAR DEMENTIA** | | | | | | | | | | | |
|  | Physical Activity | High level vs.  low level | 10,482 | 5 observational | RR | 0.62  (0.42−0.92) | 0.017 | 0.19−2.07 | 55.7 | No/No | N/A | Weak |
|  | Alcohol drinking | Light or moderate drinkers  vs. never drinkers | 9,120 | 4 observational | RR | 0.75  (0.57−0.98) | 0.037 | 0.38−1.49 | 5.2 | No/No | N/A | Weak |
|  | Antihypertensive drugs | Ever vs. never | 18,812 | 4 observational | RR | 0.64  (0.42−0.98) | 0.040 | 0.14−2.96 | 46.8 | No/No | N/A | Weak |
|  | Depression  at any stage | Diseased vs. not diseased | 13,156 | 4 observational | RR | 2.92  (1.87−4.56) | 2.5 x 10^−6^ | 1.10−7.78 | 0 | No/No | N/A | Weak |
|  | Late-life depression | Diseased vs. not diseased | 16,566 | 5 observational | OR | 2.52  (1.77−3.59) | 2.8 x 10^−7^ | 1.40−4.54 | 1.1 | No/No | N/A | Weak |
|  | Type 2 diabetes mellitus | Diseased vs. not diseased | 876,920 | 14 observational | RR | 2.28  (1.94−2.66) | 1.1 x 10^−24^ | 1.91−2.71 | 0 | No/No | N/A | Convincing |
|  | Education | Low level vs.  High level | 7,713 | 3 observational | RR | 2.75  (2.19−3.45) | 2.1 x 10^−18^ | 0.63−11.96 | 0 | No/No | N/A | Weak |
|  | Hypertension | Diseased vs. not diseased | 8,123 | 6 observational | HR | 1.59  (1.20–2.11) | 1.4 × 10^–3^ | 0.78–3.21 | 36.3 | No/No | N/A | Weak |
|  | Smoking | Ever vs never smokers | 886,794 | 8 observational | RR | 1.26  (1.05–1.50) | 0.013 | 0.79–2.00 | 43.9 | No/No | N/A | Weak |
|  | **ALZHEIMER DISEASE** | | | | | | | | | | | |
|  | Alcohol drinking | Light or moderate  drinkers  vs. never drinkers | 12,493 | 6observational | RR | 0.72  (0.61–0.86) | 2.0 x 10^−4^ | 0.44–1.18 | 56.4 | No/No | N/A | Weak |
|  | Physical activity level | High level vs. low | 20,326 | 9 observational | HR | 0.62  (0.52–0.72) | 5.0 x 10^−9^ | 0.51−0.75 | 0 | Yes/No | N/A | Highly suggestive |
|  | Depression at any age | Diseased vs. Not diseased | 46,156 | 25 observational | RR | 1.77  (1.48–2.13) | 6.0 x 10^−10^ | 0.86−3.66 | 69.6 | Yes/ Yes | N/A | Highly suggestive |
|  | Late-life depression | Diseased vs. Not diseased | 33,469 | 16 observational | RR | 1.65  (1.42–1.92) | 4.8 x 10^−11^ | 1.36−1.99 | 2.2 | No/No | N/A | Convincing |
|  | Low-frequency electromagnetic fields | Exposed vs. not exposed | 10,449,757 | 25 observational | RR | 1.74  (1.37–2.21) | 5.9 × 10^–6^ | 0.77–3.91 | 55.2 | Yes/Yes | N/A | Suggestive |
|  | Type 2 diabetes mellitus | Diseased vs. Non- diseased | 532,697 | 21 observational | RR | 1.54  (1.39–1.72) | 3.1 x 10^−15^ | 1.37−1.73 | 0 | No/No | N/A | Convincing |
|  | Vitamin C dietary intake | High vs. Low | 14,511 | 6 observational | RR | 0.85  (0.74–0.96) | 0.011 | 0.71–1.01 | 0 | No/No | N/A | Weak |
|  | Vitamin E dietary intake | High vs. low | 15,714 | 7 observational | RR | 0.80  (0.67-0.95) | 0.011 | 0.52–1.24 | 46.7 | No/No | N/A | Weak |
|  | Chlamydia pneumonia infection | Diseased vs. not diseased | 508 | 11 observational | OR | 6.0  (1.93–18.66) | 2.0 × 10^–3^ | 0.19–193.8 | 73 | No/Yes | N/A | Weak |
|  | Spirochetal infection | Diseased vs. not diseased | 555 | 13 observational | OR | 10.65  (3.40–33.42) | 5.0 × 10^–5^ | 0.41–279.54 | 51.6 | No/No | N/A | Weak |
|  | Education | Low vs.  High | 54,301 | 16 observational | RR | 1.82  (1.36–2.43) | 5.5 × 10^–5^ | 0.55–6.05 | 90.1 | No/No | N/A | Suggestive |
|  | Midlife BMI | Obese vs. normal weight | 17,712 | 5 observational | RR | 1.81  (1.22–2.69) | 3.0 × 10^–3^ | 0.52–6.29 | 63.7 | No/No | N/A | Weak |
|  | Mild traumatic  brain injury | Exposed vs. not exposed | 28,761 | 19 observational | OR | 1.40  (1.03–1.90) | 0.034 | 0.39–4.98 | 85.2 | No/No | N/A | Weak |
|  | Statins | Exposed vs. not exposed | 766,626 | 13 observational | RR | 0.72  (0.59–0.89) | 1.9 × 10^–3^ | 0.39–1.35 | 54.7 | Yes/Yes | N/A | Weak |
|  | *Herpes viridae* infection | Diseased vs. not diseased | 2,895 | 33 observational | OR | 1.38  (1.14–1.65) | 7.3 × 10^–4^ | 0.86–2.21 | 20.3 | No/Yes | N/A | Suggestive |
|  | Aspirin | Ever vs. Never | 18,656 | 11 observational | RR | 0.77  (0.63–0.95) | 0.014 | 0.42–1.42 | 55.5 | Yes/No | N/A | Weak |
|  | Non-Aspirin NSAIDS | Ever vs. Never | 18,048 | 9 observational | RR | 0.65  (0.49–0.86) | 2.3 × 10^–3^ | 0.29–1.45 | 59.1 | Yes/No | N/A | Weak |
|  | NSAIDS | Ever vs. Never | 281,491 | 16 observational | RR | 0.74  (0.64–0.86) | 6.9 × 10^–5^ | 0.45–1.22 | 70 | No/No | N/A | Suggestive |
|  | Aluminum | Exposed vs. not exposed | 10,567 | 8 observational | OR | 1.72  (1.33–2.21) | 3.1 × 10^–5^ | 1.16–2.54 | 6.2 | No/No | N/A | Suggestive |
|  | Fish intake | High vs. Low | 21,941 | 5 observational | RR | 0.88  (0.79–0.98) | 0.022 | 0.63–1.22 | 63.4 | No/No | N/A | Weak |
|  | Stroke | Diseased vs. not diseased | 14,730 | 6 observational | HR | 1.59  (1.25–2.02) | 1.7 × 10^–4^ | 1.13–2.23 | 0 | No/No | N/A | Weak |
|  | Agreeableness | High level vs. low level | 3,342 | 3 observational | HR | 0.88  (0.79–0.98) | 0.019 | 0.43–1.78 | 0 | No/No | N/A | Weak |
|  | Conscientiousness | High level vs. low level | 3,342 | 3 observational | HR | 0.77  (0.69–0.86) | 1.5 x 10^−6^ | 0.38–1.54 | 0 | Yes/Yes | N/A | Weak |
|  | Neuroticism | High level vs. low level | 5,054 | 5 observational | HR | 1.33  (1.21–1.45) | 1.9 x 10^−9^ | 1.14–1.54 | 0 | No/Yes | N/A | Weak |
|  | Openness | High level vs. low level | 3,342 | 3 observational | HR | 0.86  (0.77–0.96) | 8.3 x 10^−3^ | 0.41–1.79 | 0 | No/No | N/A | Weak |
|  | Cancer | Diseased vs. not diseased | 44,886 | 7 observational | HR | 0.62  (0.53–0.74) | 4.6 x 10^−8^ | 0.50–0.78 | 0 | Yes/No | N/A | Highly suggestive |
|  | Abbreviations: N/A = not available; RR = relative risk; OR = odds ratio; HR = hazard ratio; BMI = body mass index; NSAIDs = non-steroid anti-inflammatory drugs.  **Other environmental factors and biomarkers with non-significant associations:**   1. **Alzheimer:** Smoking; Antihypertensive drugs; Caffeine intake; Dietary intake of β carotene; Hormone replacement therapy; Calcium channel blockers; Hypertension; General anesthesia; Extraversion; Corticosteroids; Dietary Docosahexaenoic acid; Midlife BMI (underweight vs. normal weight). 2. **All types of dementia:** Caffeine intake; Social network size; Satisfaction with social network; Late-life BMI; Midlife BMI; Rural living; Fish intake. 3. **Vascular dementia:** Midlife BMI (obese vs. normal weight).   **# # #** According to the authors of the paper, *“we considered as convincing the associations that fulfilled all the following criteria: statistical significance according to random-effects model at P < 10^-6^; based on more than 1000 cases; without large between-study heterogeneity (I^2^< 50%); 95% PI excluding the null value; and no evidence of small-study effects and excess significance. Associations with >1000 cases, P < 10^-6^, and largest study presenting a statistically significant effect were graded as highly suggestive. The associations supported by >1000 cases and a significant effect at P < 10^-3^ were considered as suggestive. The remaining nominally significant associations were considered as having weak evidence”.* | | | | | | | | | | | |
| Belbasis, 2015 | **MULTIPLE SCLEROSIS** | | | | | | | | | | | |
|  | Anti-EBNA IgG  seropositivity | High vs. Low | 7,308 | 30 observational | OR | 4.46  (3.26–6.09) | 1.5 × 10^–19^ | 1.46–13.62 | 43 | No/No | 0.06 | Convincing |
|  | Anti-EBV IgG  seronegativity | High vs. Low | 2,360 | 7 observational | OR | 0.13  (0.05–0.32) | 2.0 × 10^–5^ | 0.01–1.39 | 52 | No/No | 0.05 | Weak |
|  | Anti-VCA IgG  seropositivity | High vs. Low | 6,325 | 24 observational | OR | 4.52  (2.85–7.15) | 3.4 × 10^–10^ | 0.87–23.42 | 58 | No/No | 0.24 | Weak |
|  | Infectious mononucleosis | High vs. Low | 35,655 | 18 observational | OR | 2.17  (1.97–2.39) | 3.1 × 10^–50^ | 1.96−2.41 | 0 | No/No | 0.68 | Convincing |
|  | EBV DNA in mononuclear cells and serum | High vs. Low | 729 | 6 observational | OR | 1.84  (1.02–3.30) | 0.04 | 0.39–8.63 | 49 | No/No | 0.79 | Suggestive |
|  | *Chlamydia pneumoniae*  DNA in CSF | High vs. Low | 1,690 | 19 observational | OR | 3.22  (1.20–8.59) | 0.02 | N/A | 88 | N/A | 0.46 | Weak |
|  | Intrathecal production of IgG for *Chlamydia pneumoniae* | High vs. Low | 628 | 6 observational | OR | 3.84  (1.32−11.21) | 0.01 | N/A | 55 | N/A | 0.15 | Weak |
|  | Cytomegalovirus infection | High vs. Low | 4,222 | 11 observational | OR | 0.77  (0.67−0.87) | 1.0 x 10^−6^ | N/A | N/A | N/A | N/A | N/A |
|  | Tonsillectomy at age  ≤20 years | Exposed vs. not exposed | 8,836 | 12 observational | OR | 1.32  (1.09–1.61) | 0.005 | 0.80–2.18 | 44 | No/No | 0.72 | Suggestive |
|  | Appendectomy at age  ≤20 years | Exposed vs. not exposed | 226,342 | 7 observational | OR | 1.17  (1.02–1.34) | 0.02 | 0.99–1.38 | 0 | No/No | 0.13 | Suggestive |
|  | Traumatic injury | Exposed vs. not exposed | 2,965 | 12 observational | OR | 1.41  (1.03–1.92) | 0.03 | 0.60–3.29 | 42 | No/No | 0.29 | Suggestive |
|  | Smoking | Exposed vs. not exposed | 460,671 | 14 observational | OR | 1.52  (1.39–1.66) | 1.7 × 10^–18^ | 1.37–1.68 | 0 | No/No | 0.50 | Convincing |
|  | Diphtheria vaccination | Exposed vs. not exposed | 624 | 3 observational | OR | 0.60  (0.40–0.91) | 0.02 | 0.04–8.57 | 0 | No/No | 0.88 | Suggestive |
|  | Tetanus vaccination | Exposed vs. not exposed | 4,132 | 8 observational | OR | 0.71  (0.57–0.88) | 0.002 | 0.47–1.07 | 17 | No/No | 0.15 | Suggestive |
|  | BMD in femoral neck | High vs. low | 1,420 | 10 observational | OR | 0.36  (0.21–0.61) | 1.3 × 10^–4^ | 0.06–2.26 | 81 | No/Yes | 0.20 | Weak |
|  | BMD in lumbar spine | High vs. low | 1,490 | 11 observational | OR | 0.34  (0.24–0.50) | 1.07 × 10^–8^ | 0.11–1.12 | 67 | Yes/No | 0.05§ | Weak |
|  | BMD in hip | High vs. low | 1,299 | 9 observational | OR | 0.33  (0.18–0.60) | 2.95 × 10^–4^ | 0.04–2.65 | 86 | No/No | 0.22 | Weak |
|  | Organic solvents | Exposed vs. not exposed | 1,142,801 | 15 observational | OR | 1.54  (1.03–2.29) | 0.03 | 0.37–6.39 | 77 | Yes/No | 0.06§ | Weak |
|  | Serum vitamin D levels | High vs. low | 1,836 | 11 observational | OR | 0.44  (0.24–0.78) | 0.005 | 0.05–3.70 | 89 | Yes/No | 0.05§ | Weak |
|  | Serum uric acid | High vs. low | N/A | 8 observational | OR | 0.28  (0.14–0.57) | 4.1 × 10^–4^ | 0.02–3.29 | 87 | No/No | 0.91 | Weak |
|  | Serum vitamin B_12_ | High vs. low | 730 | 8 observational | OR | 0.64  (0.44−0.93) | 0.02 | 0.26−1.60 | 38 | No/No | 0.99 | Suggestive |
|  | Serum homocysteine | High vs. low | 1,069 | 8 observational | OR | 4.57  (1.40–14.89) | 0.01 | 0.06–338 | 96 | No/No | 0.01 | Weak |
|  | Chronic cerebrospinal  venous insufficiency | Exposed vs. not exposed | 2,149 | 19 observational | OR | 8.45  (3.47–20.56) | 3.5 × 10^–6^ | 0.33–217 | 80 | No/No | 0.23 | Weak |
|  | Abbreviations: N/A = not available; OR = odds ratio; EBV = Epstein-Barr virus; VCA = viral-capsid antigen; BMD = bone mineral density; EA = early antigen; CSF = cerebrospinal fluid; EBNA = Epstein Barr nuclear antigen  §: According to this paper, *“Both criteria for existence of small-study effects fulfilled (p value for Egger’s test < 0.10, and largest study with a smaller [more conservative] effect size than random-effects summary effect size)”*.  **Other environmental factors and biomarkers with non-significant associations:**  Anti-EA IgG seropositivity; EBV DNA in CSF and brain tissue; IgG for Chlamydia pneumoniae in serum; IgG for Chlamydia pneumoniae in CSF; Tonsillectomy at age >20 years; Appendectomy at age >20 years; Adenoidectomy at age ≤ 20 years; Other surgeries at age ≤ 20 years⁋; Other surgeries at age > 20 years⁋; BCG vaccination; Hepatitis By vaccination; Influenza vaccination; MMR vaccination; Poliomyelitis vaccination; Typhoid fever vaccination; Serum folate: Dental amalgam; Asthma; Eczema; Allergic Disease; Allergic rhinitis. (^⁋^: According to the authors of the umbrella review, *“In these analyses, the component studies did not define the type of surgery”).*  **# # #** According to the authors of the paper, “*Convincing evidence criteria: more than 1000 cases, significant summary associations (p<0.001) per random-effects calculations, no evidence of small-study effects, no evidence for excess significance bias, prediction intervals not including the null, and not large heterogeneity (I^2^≤50%); suggestive evidence criteria: nominally significant summary associations (p<0.05) per random-effects calculations, no evidence of small-study effects, no evidence for excess significance bias, and not large heterogeneity(I^2^<50%); weak evidence criteria: all other risk factors with nominally significant summary associations (p<0.05); non-significant associations were p>0.05”.* | | | | | | | | | | | |
| Bellou 2016 | **PARKINSON DISEASE** | | | | | | | | | | | |
|  | Alcohol intake | High vs.  Low | 9,994^¤^ | 33 observational | RR | 0.75  (0.66–0.85) | 5.0 × 10^–6^ | 0.44–1.25 | 52.3 | Yes/Yes | N/A | III |
|  | Coffee drinking | High vs.  Low | 5,801^¤^ | 19 observational | RR | 0.67  (0.58–0.76) | 3.4 × 10^–9^ | 0.45–1.00 | 42.9 | Yes/Yes | N/A | III |
|  | Smoking | Ever vs. never smokers | 19,537^¤^ | 67 observational | RR | 0.64  (0.60–0.69) | 1.3 × 10^–37^ | 0.45–0.92 | 49.6 | Yes/Yes | N/A | II |
|  | Physical activity | High vs.  Low level | 1,348^¤^ | 5 observational | HR | 0.66  (0.57–0.78) | 3.0 × 10^–7^ | 0.55–0.80 | 0 | No/No | N/A | I |
|  | Welding | Exposed vs. not exposed | 8,198^¤^ | 9 observational | RR | 0.86  (0.80–0.92) | 3.0 × 10^–5^ | 0.79–0.94 | 0 | No/No | N/A | III |
|  | Hydrocarbon exposure | Exposed vs not exposed | 4,483^¤^ | 14 observational | OR | 1.36  (1.13–1.63) | 0.001 | 0.88–2.08 | 28.1 | Yes/Yes | N/A | IV |
|  | Farming | Exposed vs. not exposed | 9,533^¤^ | 38 observational | OR | 1.30  (1.16–1.46) | 5.7 × 10^–6^ | 0.86–1.98 | 37.3 | No/No | N/A | III |
|  | Organic solvents | Exposed vs. not exposed | 3,811^¤^ | 18 observational | OR | 1.22  (1.01–1.47) | 0.036 | 0.72–2.08 | 43.6 | Yes/Yes | N/A | IV |
|  | Pesticides | Exposed vs. not exposed | 7,151^¤^ | 39 observational | OR | 1.62  (1.40–1.88) | 1.1 × 10^–10^ | 0.81–3.23 | 63.7 | Yes/Yes | N/A | III |
|  | Rural living | Exposed vs. not exposed | 4,306^¤^ | 31 observational | OR | 1.32  (1.18–1.48) | 1.7 × 10^–6^ | 0.84–2.10 | 78.6 | Yes/Yes | N/A | III |
|  | Well water drinking | Exposed vs not exposed | 5,037^¤^ | 28 observational | RR | 1.21  (1.05–1.40) | 0.011 | 0.66–2.21 | 70.6 | No/Yes | N/A | IV |
|  | Vitamin E intake | High vs. Low | 936^¤^ | 7 observational | OR | 0.81  (0.67–0.98) | 0.028 | 0.63–1.04 | 0 | No/No | N/A | IV |
|  | Dairy products intake | High vs.  Low | 1,083^¤^ | 7 observational | RR | 1.40  (1.20–1.63) | 2.4 × 10^–5^ | 1.08–1.81 | 8.2 | No/No | N/A | III |
|  | Carbohydrate intake | High vs.  Low | 1,482^¤^ | 8 observational | RR | 1.24  (1.05–1.48) | 0.014 | 1.00–1.54 | 0 | No/No | N/A | IV |
|  | Energy intake | High vs.  Low | 1,415^¤^ | 8 observational | RR | 1.39  (1.01–1.92) | 0.042 | 0.50–3.90 | 83.8 | Yes/Yes | N/A | IV |
|  | Constipation | Exposed vs. not exposed | 11,242^¤^ | 9 observational | RR | 2.30  (2.02–2.63) | 3.5 × 10^–35^ | 1.76–2.96 | 18.2 | No/No | N/A | I |
|  | Head injury | Exposed vs. not exposed | 35,799^¤^ | 22 observational | OR | 1.55  (1.33–1.81) | 2.2 × 10^–8^ | 0.93–2.58 | 61 | No/No | N/A | II |
|  | Anxiety or Depression | Exposed vs. not exposed | 16,211^¤^ | 13 observational | RR | 1.86  (1.64–2.10) | 2.6 × 10^–22^ | 1.30–2.66 | 67.7 | No/No | N/A | II |
|  | Hypertension | Exposed vs. not exposed | 5,993^¤^ | 12 observational | RR | 0.75  (0.61–0.90) | 0.003 | 0.40–1.40 | 76.4 | No/Yes | N/A | IV |
|  | α-synuclein in CSF | High vs. low | 850^¤^ | 11 observational | OR | 0.29  (0.13−0.62) | 0.002 | 0.02−5.19 | 91.7 | No/Yes | N/A | IV |
|  | Serum Vitamin D | High vs. low | 1,008^¤^ | 7 observational | OR | 0.16  (0.05–0.50) | 0.002 | 0.003–10.09 | 97.7 | Yes/Yes | N/A | IV |
|  | Nigral volume | High vs. low | 193^¤^ | 8 observational | OR | 0.31  (0.17–0.55) | 8.3 × 10^–5^ | 0.06–1.46 | 47.4 | No/No | N/A | IV |
|  | Serum urate | High vs low | 594^¤^ | 6 observational | RR | 0.65  (0.43–0.97) | 0.034 | 0.23–1.82 | 42.1 | No/No | N/A | IV |
|  | Serum uric acid | High vs. Low | 1,217^¤^ | 6 observational | OR | 0.39  (0.27–0.57) | 6.8 × 10^–7^ | 0.13–1.22 | 75.9 | No/No | N/A | II |
|  | Serum vitamin B_12_ | High vs Low | 735^¤^ | 10 observational | OR | 0.50  (0.40–0.63) | 4.7 × 10^–9^ | 0.31–0.82 | 23.8 | No/Yes | N/A | IV |
|  | Retinal nerve fiber layer thickness | High vs. low | 644^¤^ | 13 observational | OR | 0.40  (0.24−0.66) | 3.5 x 10^−4^ | 0.06−2.55 | 81 | No/Yes | N/A | IV |
|  | Bone mineral density in femoral neck | High vs. low | 561^¤^ | 8 observational | OR | 0.25  (0.09−0.66) | 0.005 | 0.01−8.76 | 95.6 | No/No | N/A | IV |
|  | Bone mineral density in hip | High vs. low | 401^¤^ | 6 observational | OR | 0.55  (0.38−0.80) | 0.002 | 0.18−1.66 | 61.8 | No/Yes | N/A | IV |
|  | Bone mineral density in lumbar spine | High vs. low | 611^¤^ | 9 observational studies | OR | 0.29  (0.16−0.54) | 7.8 x 10^−5^ | 0.03−2.60 | 89 | No/Yes | N/A | IV |
|  | Non-aspirin NSAIDS | Exposed vs not exposed | 3,967^¤^ | 7 observational | RR | 0.85  (0.77−0.94) | 0.002 | 0.74−0.97 | 0.1 | Yes/No | N/A | IV |
|  | Ibuprofen use | Exposed vs not exposed | 2,170^¤^ | 5 observational | RR | 0.73  (0.62−0.85) | 6.6 x 10^−5^ | 0.57−0.94 | 0 | No/No | N/A | III |
|  | Beta-blockers | Exposed vs not exposed | 5,774^¤^ | 3 observational | RR | 1.28  (1.19−1.39) | 5.0 x 10^−10^ | 0.77−2.13 | 0 | No/No | N/A | II |
|  | Calcium channel blockers | Exposed vs not exposed | 6,966^¤^ | 5 observational | RR | 0.78  (0.67−0.90) | 7.0 x 10^−4^ | 0.55−1.11 | 25.7 | No/No | N/A | III |
|  | Statins | Exposed vs not exposed | 15,102^¤^ | 8 observational | RR | 0.77  (0.64−0.92) | 0.004 | 0.47−1.27 | 62.9 | Yes/No | N/A | IV |
|  | Abbreviations: N/A = not available; RR = relative risk; OR = odds ratio; HR = hazard ratio; NSAIDS = non-steroidal anti-inflammatory drugs; CSF = cerebrospinal fluid  ^¤^Number of cases.  **Other environmental factors and biomarkers with non-significant associations:**  Outdoor work; Tea drinking; Extremely low frequency magnetic fields; Manganese Exposure.  Vitamin C intake; Vitamin D supplementation; Folate intake; Vitamin B_12_ intake; Vitamin B_6_ intake; Lutein intake; Lycopene intake; Vitamin A intake; α-Carotene intake; β-Carotene intake; β-Cryptoxanthin intake; Cholesterol intake; Protein intake; Total fat intake.  Diabetes mellitus; Melanoma; Cancer; Gastric ulcer; Oophorectomy; Gout.  Aspirin; Acetaminophen; General anesthesia; Oral contraceptive; Hormone replacement.  BMI (BMI ≥ 30 vs. BMI < 25); BMI (BMI ≥ 30 vs. 25 ≤ BMI < 30); BMI (25 ≤ BMI < 30 vs. BMI < 25); BMI (per 5 kg/m^2^ increase); Serum cholesterol; Copper in plasma; Copper in CSF; Serum copper; Iron in CSF; Serum iron; Serum folate; Bone mineral density in trochanter.  **# # #** According to the authors of the paper, “ *we used the following categories: Convincing evidence (Class I) required >1000 cases, highly significant summary associations (p < 10^-6^ by random-effects), no evidence of small-study effects, no evidence of excess significance bias, 95% prediction interval not including the null and not large heterogeneity (I^2^< 50%). Highly suggestive evidence (Class II)required >1000 cases, highly significant summary associations(p < 10^-6^ by random effects) and largest study with 95% CI excluding the null value. Suggestive evidence (Class III) required only >1000 cases and p < 0.001 by random effects. All other risk factors with nominally significant summary associations (p < 0.05)were coined as having weak evidence (Class IV). Non-significant associations were those with p > 0.05”.* | | | | | | | | | | | |

**Table S2.** Methodological quality of included umbrella reviews based on the AMSTAR criteria and score.

| **Author, year** | **Q1** | **Q2** | **Q3** | **Q4** | **Q5** | **Q6** | **Q7** | **Q8** | **Q9** | **Q10** | **Q11** | **Total** |
| --- | --- | --- | --- | --- | --- | --- | --- | --- | --- | --- | --- | --- |
| **Dinu, 2018** | No | Yes | Yes | Yes | No | Yes | Yes | Yes | Yes | Yes | No | 8 |
| **Poole, 2017** | No | Yes | Yes | Yes | No | Yes | Yes | Yes | Yes | No | No | 7 |
| **Grosso, 2017** | No | Yes | Yes | Yes | No | Yes | Yes | Yes | Yes | Yes | No | 8 |
| **Li, 2017** | No | Yes | Yes | Yes | No | Yes | Yes | Yes | Yes | Yes | No | 8 |
| **Belbasis, 2016** | No | Yes | Yes | Yes | No | Yes | Yes | Yes | Yes | Yes | No | 8 |
| **Bellou, 2016** | No | Yes | Yes | Yes | No | Yes | Yes | Yes | Yes | Yes | No | 8 |
| **Veronese, 2018** | No | Yes | Yes | Yes | No | Yes | Yes | Yes | Yes | Yes | No | 8 |
| **Posadzki, 2018** | Yes | Yes | Yes | Yes | No | Yes | Yes | Yes | Yes | Yes | No | 9 |
| **Bellou, 2017** | No | Yes | Yes | Yes | No | Yes | Yes | Yes | Yes | Yes | No | 8 |
| **McRae, 2017** | No | Yes | Yes | Yes | No | Yes | Yes | Yes | Yes | Yes | No | 8 |
| **Belbasis, 2015** | No | Yes | Yes | Yes | No | Yes | Yes | Yes | Yes | Yes | No | 8 |
| **Theodoratou, 2014** | No | Yes | Yes | Yes | No | Yes | Yes | Yes | Yes | Yes | No | 8 |
| **Galbete, 2018** | No | Yes | Yes | Yes | No | Yes | Yes | Yes | Yes | Yes | No | 8 |
| **Veronese, 2019** | No | Yes | Yes | Yes | No | Yes | Yes | Yes | Yes | Yes | No | 8 |

**AMSTAR:** A Measurement tool to Assess Systematic Reviews [please see Ref.:18]; # Q1: *A-priori* design; Q2: Duplicate study selection and data extraction; Q3: Search comprehensiveness; Q4: Inclusion of gray literature; Q5: Included and excluded studies provided; Q6: Characteristics of the included studies provided; Q7: Scientific quality of the primary studies assessed and documented; Q8: Scientific quality of included studies used appropriately in formulating conclusions; Q9: Appropriateness of methods used to combine studies’ findings; Q10: Likelihood of publication bias was assessed; Q11: Conflict of interest—potential sources of support were clearly acknowledged in both the systematic review and the included studies.
