# Supplementary material for: Non-genetic risk and protective factors and biomarkers for neurological disorders: a meta-umbrella systematic review of umbrella reviews
Source: BMC Med. 2021 Jan 13;19:6. doi: 10.1186/s12916-020-01873-7 (PMC7805241; doi:10.1186/s12916-020-01873-7)
Supplement: Supplementary file 3 — Additional file 3. Updated search of umbrella reviews. [file 12916_2020_1873_MOESM3_ESM.docx]

**Additional File 3. Updated search of umbrella reviews**

We performed an updated, supplementary search in PubMed on umbrella reviews, using “umbrella review” [ti] as search string, and publication dates from September 21^st^, 2018, and until January 1^st^, 2020. This search returned a good number of results. After independent analysis by four reviewers (AFAM, ED, VE, and GPC), 23 umbrella reviews were considered of potential relevance to our research questions (see references 46, 84, 85, 183-202 in the main manuscript file).
